# Inhibiting TRIM21 Neddylation Rejuvenates Oocyte Quality in PCOS by Regulating Ubiquitination of CPT1A

**DOI:** 10.34133/research.1223

**Published:** 2026-04-03

**Authors:** Xinni Na, Jinchi Liu, Yinan Tan, Lingbo Meng, Cuishan Guo, Na Zuo, Wanlin Dai, Ruiting Cong, Bowen Zhang, Wanting Shi, Jia Hu, Junzhi Liang, Shuang Wei, Zhongyu Zhao, Jing Chen, Xinbo Qiao, Da Li

**Affiliations:** ^1^ Department of Obstetrics and Gynecology, Shengjing Hospital of China Medical University, Shenyang, China.; ^2^ Center of Reproductive Medicine, Department of Obstetrics and Gynecology, Shengjing Hospital of China Medical University, Shenyang, China.; ^3^ NHC Key Laboratory of Advanced Reproductive Medicine and Fertility (China Medical University), National Health Commission, Shenyang, China.

## Abstract

Polycystic ovary syndrome (PCOS) is a prevalent endocrine and metabolic disorder affecting women of childbearing age. Infertility caused by ovulatory disorders and low follicle quality is an urgent problem. Abnormal fatty acid oxidation (FAO) contributes critically to the etiology and progression in PCOS. However, the potential mechanism by which FAO affects follicular development in PCOS patients is unclear. Here, we demonstrate that increased expression of tripartite motif-containing protein 21 (TRIM21) (an E3 ubiquitin ligase) in the prenatal anti-Müllerian hormone PCOS mouse model leads to abnormal FAO in ovarian granulosa cells. Functionally, knocking down TRIM21 increased mitochondrial membrane potential homeostasis and improved fatty acid-dependent aerobic respiration and oxidative phosphorylation functions. TRIM21 regulated the ubiquitination and degradation of carnitine palmitoyltransferase 1A (CPT1A), a key enzyme for FAO, at the K161 site. TRIM21 is regulated by the E2 ubiquitin ligase ubiquitin conjugating enzyme E2 M (UBE2M), which enhances its protein expression and ubiquitination by TRIM21 neddylation. MLN4924 (a neddylation inhibitor) reversed the ubiquitination degradation of CPT1A by inhibiting TRIM21 neddylation. The phenotype of PCOS mice treated with MLN4924 was alleviated, and the maturation of oocytes and embryonic development improved. Together, these findings indicate that TRIM21-CPT1A plays an indispensable role in FAO of granulosa cells, and inhibiting TRIM21 neddylation may be a therapeutic strategy to improve abnormal follicular development in PCOS.

## Introduction

Polycystic ovary syndrome (PCOS) is one of the most common ovarian diseases affecting female fertility, with a global prevalence of 10% to 13% [1]. According to the Rotterdam criteria, the diagnosis of PCOS requires 2 of the following characteristics: hyperandrogenism, irregular menstruation (oligo-ovulation or anovulation), and polycystic ovarian morphology. The 2023 International PCOS Assessment and Management Guidelines recommend anti-Müllerian hormone (AMH) as an alternative to transvaginal ultrasound for evaluating polycystic ovarian morphology [2]. Accumulating evidence indicates that metabolic dysfunction is a principal driver of PCOS onset and progression and can be transmitted across generations [3]. Meanwhile, ovulation disorders and abnormal follicular development are important factors in infertility in women with PCOS. Although assisted reproductive technology can compensate for this undesirable reproductive outcome to a certain extent [4], the quality of oocytes collected from patients is often poor, resulting in a high cancellation rate and a low fertilization rate [5]. Therefore, improving the quality of follicles in PCOS patients and enhancing fertility is the focus of current research.

Fatty acid oxidation (FAO) is the process of breaking down medium-chain fatty acids into acyl-coenzyme A to produce reducing equivalents (nicotinamide adenine dinucleotide [reduced form] or flavin adenine dinucleotide [reduced form]) in the mitochondrial matrix through β-oxidation to provide electrons, ultimately driving oxidative phosphorylation (OXPHOS) to produce adenosine triphosphate (ATP) through the respiratory chain complex. Primordial to preovulatory follicle development is a high-energy-demand process. The oocyte acquires its developmental competence within the ovarian follicle, a structure enclosed by somatic cells that are predominantly granulosa cells (GCs) [6,7]. GCs provide oocytes with essential nutrients and metabolic precursors through transzonal projection, enabling them to acquire developmental capacity and prepare for successful fertilization following meiotic maturation [8,9]. Moreover, FAO in GCs provides the necessary substrates for steroid synthesis to support follicular maturation along with sufficient metabolic intermediates to drive cytoskeleton reorganization and enzyme activation when the follicle ruptures and the cumulus–oocyte complex (COC) is expelled [10,11]. It has been reported that the down-regulation of key FAO proteins in the GCs of PCOS patients results in an impaired superovulation response [12], and mitochondrial dysfunction is associated with fertilization failure and blastocyst formation [13]. Although studies have suggested that FAO plays an essential role in regulating folliculogenesis and ovulation, its specific regulatory mechanism in the ovarian GCs of patients with PCOS has not been systematically elucidated, especially the process of regulating key enzymes and signaling pathways at the level of protein posttranslational modifications, for which direct evidence is still lacking.

Tripartite motif (TRIM) proteins comprise one of the largest subfamilies of RING-type E3 ubiquitin ligases, regulating essential biological processes such as signal transduction, innate immunity, and tumorigenesis [14]. Emerging research has highlighted the notable role of TRIM family proteins in metabolic homeostasis. Various TRIM members are implicated in regulating key metabolic pathways, including lipid metabolism, glucose homeostasis, and insulin signaling, with their dysregulation linked to metabolic disorders such as obesity, nonalcoholic fatty liver disease, and insulin resistance [15–17]. TRIM21 is a member of the TRIM family that can catalyze the synthesis of ubiquitin chains linked to Lys48 (K48) and Lys63 (K63), thereby generating ubiquitin-tagged substrates for degradation by the 26S proteasome [18]. In diet-induced obese mice, TRIM21 promotes fatty acid synthase (FASN) degradation through direct protein–protein interaction-mediated ubiquitination, which contributes to hepatic lipid metabolism [19], positioning it as a potential regulator at the intersection of metabolism and disease.

Our group has previously focused on glucose metabolism mediated by TRIM family members [20] and the necessity of fatty acids for in vitro embryo development and implantation [21]. By sequencing the transcriptomes of the ovaries of prenatal mice exposed to AMH, we found that TRIM21 was expressed at elevated levels in mouse ovarian GCs. Further proteomics analyses suggested that TRIM21 is enriched in biological pathways related to FAO. We speculate that TRIM21-mediated GC FAO may be a key link in the progression of PCOS. In this study, we investigated the mechanism by which TRIM21 regulates the homeostasis of key enzymes involved in FAO and examined whether this mechanism affects the quality of oocytes. We verified that targeting TRIM21 could become a potential therapeutic measure for improving the fertility of PCOS patients.

## Results

### High expression of TRIM21 in ovarian GCs of PCOS mice

**Fig. 1.**
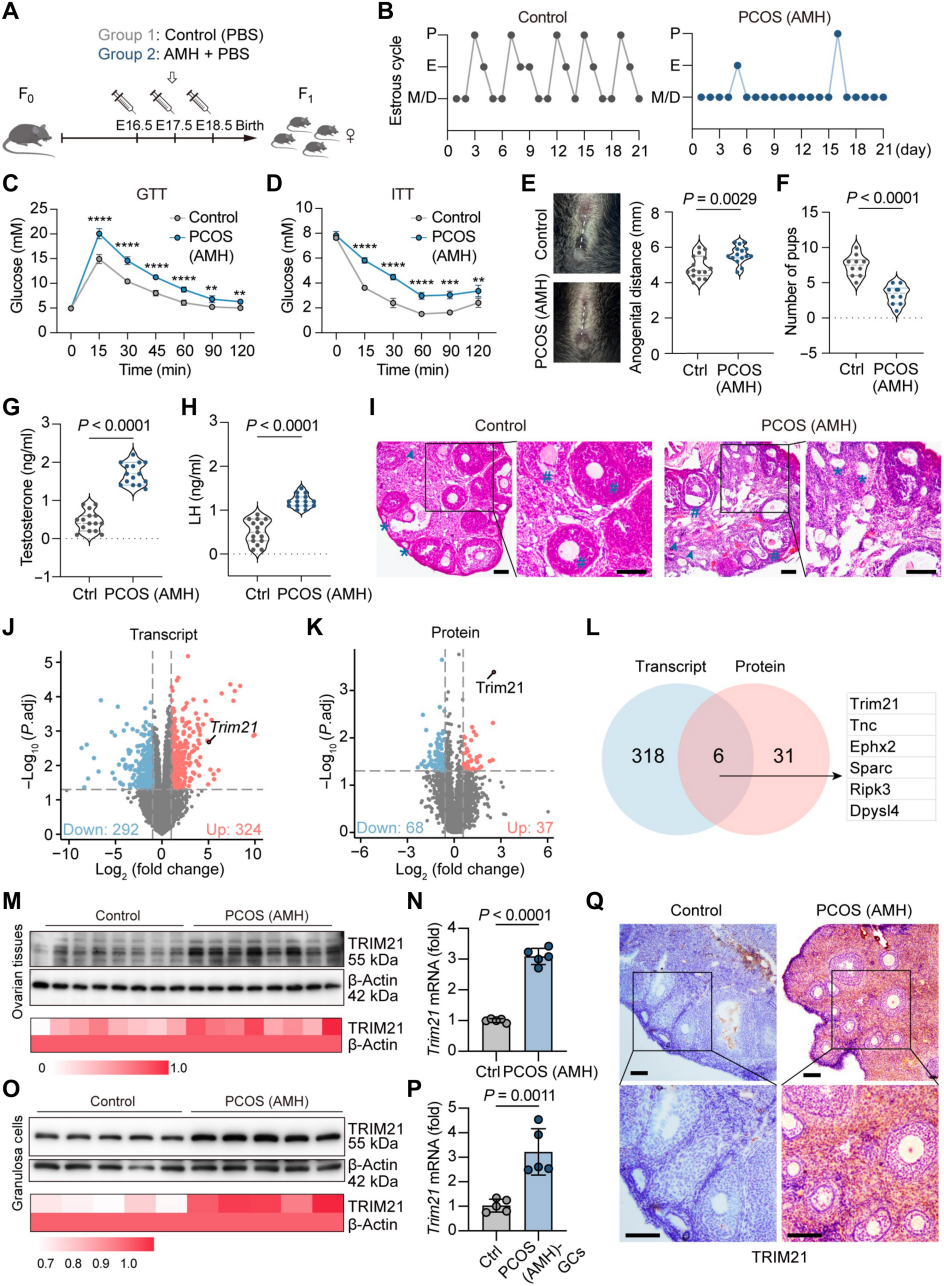
High expression of tripartite motif-containing protein 21 (TRIM21) in ovarian granulosa cells (GCs) of polycystic ovary syndrome (PCOS) mice. (A) Schematic illustration showing the induction of PCOS by prenatal anti-Müllerian hormone (PAMH) treatment. (B) Estrous cycle of adult female mice for 21 consecutive days (*n* = 5). (C and D) Glucose tolerance test and insulin tolerance test after 16 h of fasting and 4 h of fasting in adult female mice (*n* = 5). (E) Anogenital distance in adult female mice (*n* = 15). (F) Number of pups per birth (*n* = 10). (G and H) Testosterone and luteinizing hormone (LH) levels of serum (*n* = 15). (I) Hematoxylin and eosin (H&E) staining of ovaries showing primordial follicles (*), growing follicles (#), and atretic follicles (arrows; scale bars, 100 μm). (J and K) Volcano plots showing differentially expressed proteins and genes in the ovaries of control and PAMH F_1_ mice (up-regulated, red; down-regulated, blue; *n* = 3). (L) Up-regulated proteins and genes determined by proteomic and transcriptomic analysis. (M and N) Western blot (WB) and reverse transcription polymerase chain reaction (RT-PCR) analysis of TRIM21 in mouse ovarian tissues. (O and P) WB and RT-PCR analysis of TRIM21 in mouse GCs. (Q) Immunohistochemical staining of TRIM21 in mouse ovarian tissues (scale bars, 100 μm). Data are expressed as means ± SD, and each symbol represents a biologically independent mouse. Significance was calculated by 1-way analysis of variance (ANOVA) multiple comparison test. Blood glucose analysis between groups (C and D) was determined by 2-way ANOVA and multiple comparison test. **P < 0.01; ***P < 0.001; ****P < 0.0001.

To explore the cause of low fertility in PCOS women, we established prenatal AMH (PAMH) F_1_ mice with a PCOS phenotype by prenatal exposure to high AMH (Fig. 1A). Compared with the control group, PAMH F_1_ mice showed a dysregulated estrous cycle (Fig. 1B and Fig. S1A), impaired glucose tolerance, and decreased insulin sensitivity (Fig. 1C and D) but no significant weight changes (Fig. S1B), which means that AMH mice are more consistent with the lean PCOS phenotype. The anogenital distance (AGD) of PAMH F_1_ mice was significantly different from that of the control group, consistent with the characteristics of androgen exposure (Fig. 1E). The fertility of the PAMH F_1_ females were also significantly affected, as shown by a decrease in the number of litters (Fig. 1F and Fig. S1C). The levels of testosterone and luteinizing hormone in serum were significantly increased (Fig. 1G and H). Ovarian histological analysis showed that there was no significant difference in ovarian structure between the PAMH F_1_ and control mice, but the proportion of mature follicles was significantly reduced (Fig. 1I). Meanwhile, we obtained another PCOS-like mouse model by intraperitoneal injection of dihydrotestosterone (DHT) to acquire high androgen and metabolic disorders similar to PCOS (Fig. S1D). The PCOS characteristics of the DHT-treated mice were consistent with those of the PAMH F_1_ mice (Figs. S1E to G and S2A and B), but the DHT-treated mice showed significant weight gain, and ovarian tissue analysis showed characteristic polycystic ovary morphology and a decrease in the number of corpora lutea (Fig. S2C and D).

We removed the ovaries of PAMH F_1_ mice and control mice and used proteomic (label-free liquid chromatography-tandem mass spectrometry; Table S1) and transcriptomic multiomics sequencing to analyze the differential expression of genes and proteins (Table S2). Compared with the control group, the ovaries of mice in the PAMH F_1_ group had 324 up-regulated genes in the transcriptome and 37 up-regulated proteins in the proteome (cutoff value was a 2-fold change, *P* < 0.05; Fig. 1J and K). After integrating the results of the 2 sequencing analyses, 6 genes or proteins were significantly up-regulated in both analyses, among which Trim21 showed the highest fold difference (Fig. 1L). TRIM21 belongs to the tripartite motif protein family and has E3 ubiquitin ligase activity. Research on TRIM21 has focused on its role in tumors and autoimmune diseases [22,23], and there are no reports of TRIM21 in PCOS. By western blotting and reverse transcription polymerase chain reaction (RT-PCR), the expression of TRIM21 was increased in the ovarian tissue of PAMH F_1_ and DHT mice (Fig. 1M and N and Fig. S2E and F). Next, we isolated and cultured the ovarian GCs of both control and PCOS mice (Fig. S2G) and then analyzed them by western blot and RT-PCR. Compared with the control group, the expression of TRIM21 in the PAMH F_1_ and DHT mice was increased (Fig. 1O and P). Immunohistochemistry of the ovarian tissue also indicated that TRIM21 was up-regulated in the GCs of the PCOS group (Fig. 1Q and Fig. S2H). We performed PCR assays again in the follicular fluid of PCOS women and obtained the same conclusion (Fig. S2I). Collectively, TRIM21 is highly expressed in GCs of PCOS mice and is highly associated with PCOS disease.

### TRIM21 is associated with FAO in ovarian GCs

**Fig. 2.**
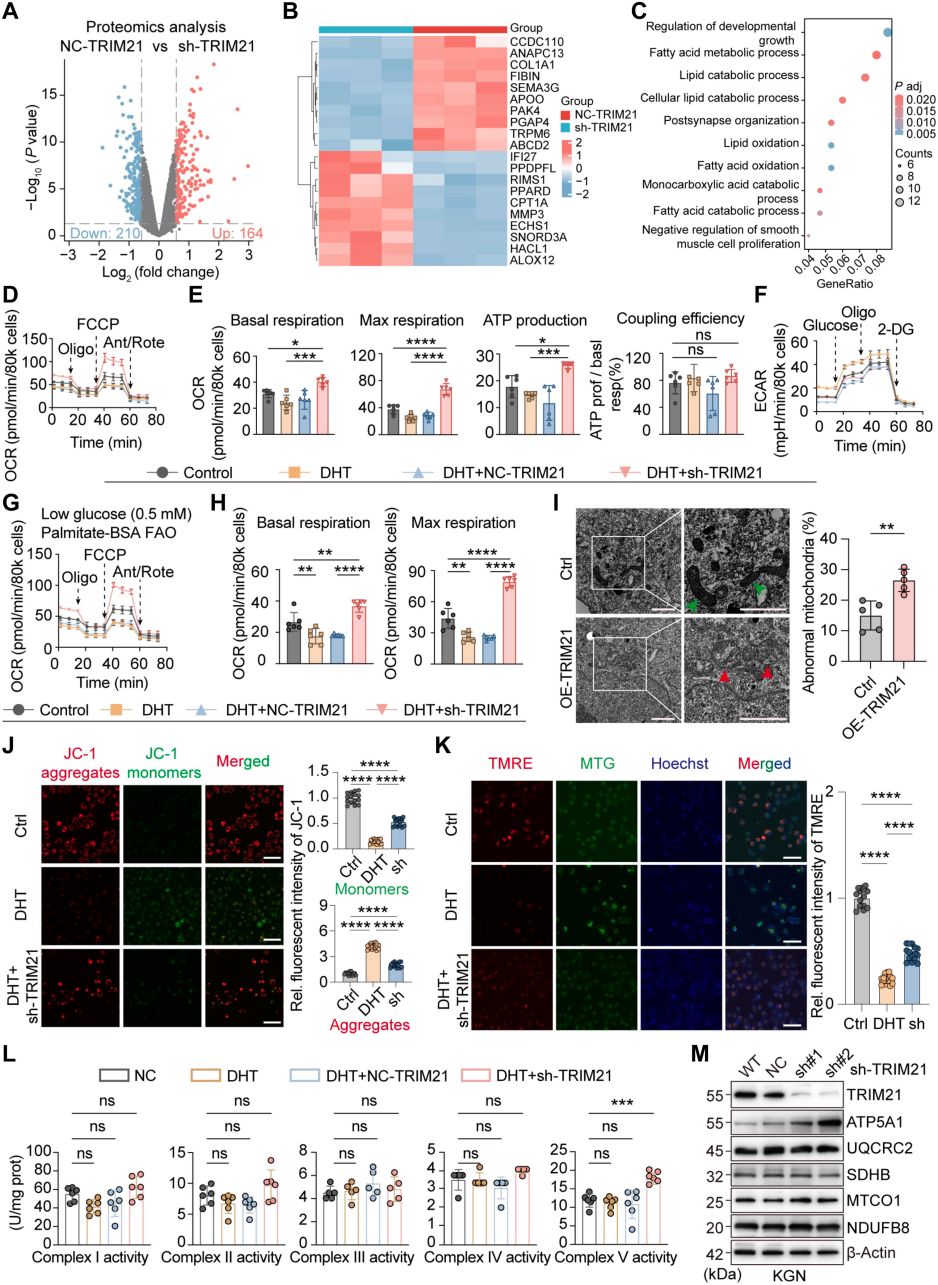
Tripartite motif-containing protein 21 (TRIM21) is associated with fatty acid oxidation (FAO) in ovarian granulosa cells. (A) Volcano plot for the significant (*P* < 0.05) alterations in protein expression induced by TRIM21 knockdown (*n* = 3). (B) Heatmap plot showing differentially expressed proteins (*n* = 3). (C) Gene Ontology (GO) enrichment analysis of the proteomic sequencing. (D and E) Evaluation of the mitochondrial oxidative phosphorylation (OXPHOS) function by oxygen consumption rate (OCR) in KGN cells, including basal respiration, maximum respiration, adenosine triphosphate (ATP) production, and coupling efficiency (*n* = 6). (F) Evaluation of the glycolysis function by extracellular acidification rate (ECAR) in KGN cells (*n* = 3). (G and H) Evaluation of FAO-dependent mitochondrial function by OCR in KGN cells, including basal respiration and maximum respiration (*n* = 6). (I) transmission electron microscopy (TEM) analysis of mitochondrial morphology in KGN cell. Green arrows indicate normal mitochondria, and red arrows indicate abnormal mitochondria (*n* = 5; scale bars, 2 μm). (J) Representative images for JC-1 (*n* = 15; scale bar, 50 μm) and ratio of the fluorescence intensities in KGN cells. (K) Representative images for tetramethylrhodamine ethyl ester perchlorate (TMRE) (*n* = 15; scale bar, 50 μm) and ratio of the fluorescence intensities in KGN cells. (L) Activity of mitochondrial complexes I to V in KGN cells (*n* = 6). (M) Western blot (WB) analysis of OXPHOS subunits (complex I-NDUFB8, complex II-SDHB, complex III-UQCRC2, complex IV-MTCO1, and complex V-ATP5A1) of KGN cells. Data are expressed as means ± standard error of the mean (SEM). ns, not significant; *P < 0.05; **P < 0.01; ***P < 0.001; ****P < 0.0001.

Previous studies have found that TRIM21 affects metabolic reprogramming in an inflammatory environment and is involved in regulating mitochondrial membrane potential and autophagy levels [24,25]. To understand the role of TRIM21 in PCOS, we performed proteomic sequencing of TRIM21 knockdown KGN cells by treating cells with short hairpin RNA (shRNA) negative control (sh-NC) and sh-TRIM21 (Table S3). There were 210 down-regulated proteins and 164 up-regulated proteins after TRIM21 knockdown (cutoff value was a 1.5-fold change, *P* < 0.05; Fig. 2A and B). Considering that TRIM21 has E3 ubiquitin ligase activity, TRIM21 may down-regulate protein expression. Therefore, we focused on the proteins that were significantly up-regulated after TRIM21 knockdown and performed Gene Ontology (GO) functional enrichment analysis on these differential proteins. Among the top 10 significantly enriched pathways, there were many pathways related to lipid metabolism, including lipid degradation and FAO, indicating that TRIM21 is closely related to lipid metabolism in ovarian GCs (Fig. 2C).

To verify the above results, we performed a series of bioenergetics measurements in KGN cells using a Seahorse XF extracellular flux analyzer. The mitochondrial function was assessed by testing the oxygen consumption rate (OCR) at baseline and after the sequential addition of oligomycin (an inhibitor of ATP synthesis), carbonyl cyanide-trifluoromethoxy phenylhydrazone (FCCP, a mitochondrial uncoupler), and antimycin/rotenone (an inhibitor of complex I and III; Fig. 2D). After the addition of DHT to simulate PCOS GCs, the basal respiration was reduced, but there was no difference in comparison with the NC group, while the basal respiration of DHT+sh-TRIM21 cells was significantly improved compared with the NC group. The maximum respiration of DHT+sh-TRIM21 cells was significantly elevated to even higher than that of the NC group, as measured by the addition of FCCP. Oligomycin-dependent mitochondrial ATP-linked respiration also indicated an increase in the sh-TRIM21 group. The coupling efficiency (the relationship between ATP generation and basal respiration) remained relatively constant among the groups (Fig. 2E). The basal respiration value and maximum respiration were significantly decreased in the GCs after overexpression of TRIM21 compared with the NC group (Fig. S3A and B). Next, glycolysis was determined by measuring the changes in the extracellular acidification rate (ECAR) after the addition of glucose, oligomycin, and 2-deoxy-d-glucose in sequence. The results showed that glycolysis was up-regulated in the GCs of DHT+sh-TRIM21 but not remarkably different (Fig. 2F and Fig. S3C), probably to compensate for the defect of OXPHOS. The glycolysis and glycolytic capacity in the TRIM21-overexpression group were not significantly different from those in the NC group (Fig. S3D and E). To verify the connection between FAO and OXPHOS, we measured the FAO-dependent respiration rate in DHT+sh-TRIM21 cells. FAO-dependent respiration was determined by measuring the OCR in culture medium with minimal glucose content and palmitate-bovine serum albumin (BSA) as a long-chain fatty acid substrate. In the sh-TRIM21 group, the basal and maximum respiration rates were significantly increased (Fig. 2G and H), which supports the association between TRIM21 and FAO. We also found that the mitochondrial morphology in the GCs of the TRIM21-overexpression group also changed compared with the control. By transmission electron microscopy (TEM), swollen mitochondria appeared in the overexpression group cells (Fig. 2I), and the morphological abnormalities were consistent with functional abnormalities.

FAO occurs in the mitochondrial matrix, and the mitochondrial membrane potential (ΔΨm) is a key indicator of mitochondrial function. It is driven by redox reactions promoted by the electron transport chain complex and ultimately leads to ATP synthesis through ATP synthase (complex V) [26,27]. DHT was added to KGN cells to simulate the phenotype of GCs in PCOS patients. Analysis of mitochondrial function by JC-1 and tetramethylrhodamine ethyl ester perchlorate (TMRE) staining showed that the mitochondrial membrane potential decreased in the DHT group, while it increased in the sh-TRIM21 group and was remarkably different from the NC group (Fig. 2J and K). Mitochondrial energy production mainly depends on OXPHOS complexes I to V. The complex V of sh-TRIM21-treated GCs increased (Fig. 2L), indicating that the total amount of mitochondrial respiratory electron transfer activity increased, while complexes I to IV did not show significant differences (Fig. 2L). This may be because the respiratory chain complexes react to the maximum catalytic activity of individual complex enzymes, while Seahorse XF analysis detects dynamic mitochondrial function in living cells. When TRIM21 was overexpressed, complexes I to V showed a downward trend, but there was no statistical difference (Fig. S3F). Western blotting of OXPHOS-related proteins in KGN and COV434 cell lines indicated no statistically significant difference in complexes I to IV after sh-TRIM21 treatment compared with control, and only the expression of ATP synthase F1 subunit alpha (ATP5A1) was significantly increased (Fig. 2M and Fig. S3G), which is consistent with the results of the respiratory chain complexes. In summary, these measurements confirm that TRIM21 is involved in FAO, mitochondrial membrane potential homeostasis, and OXPHOS in ovarian GCs.

### TRIM21 regulates FAO in GCs through CPT1A

**Fig. 3.**
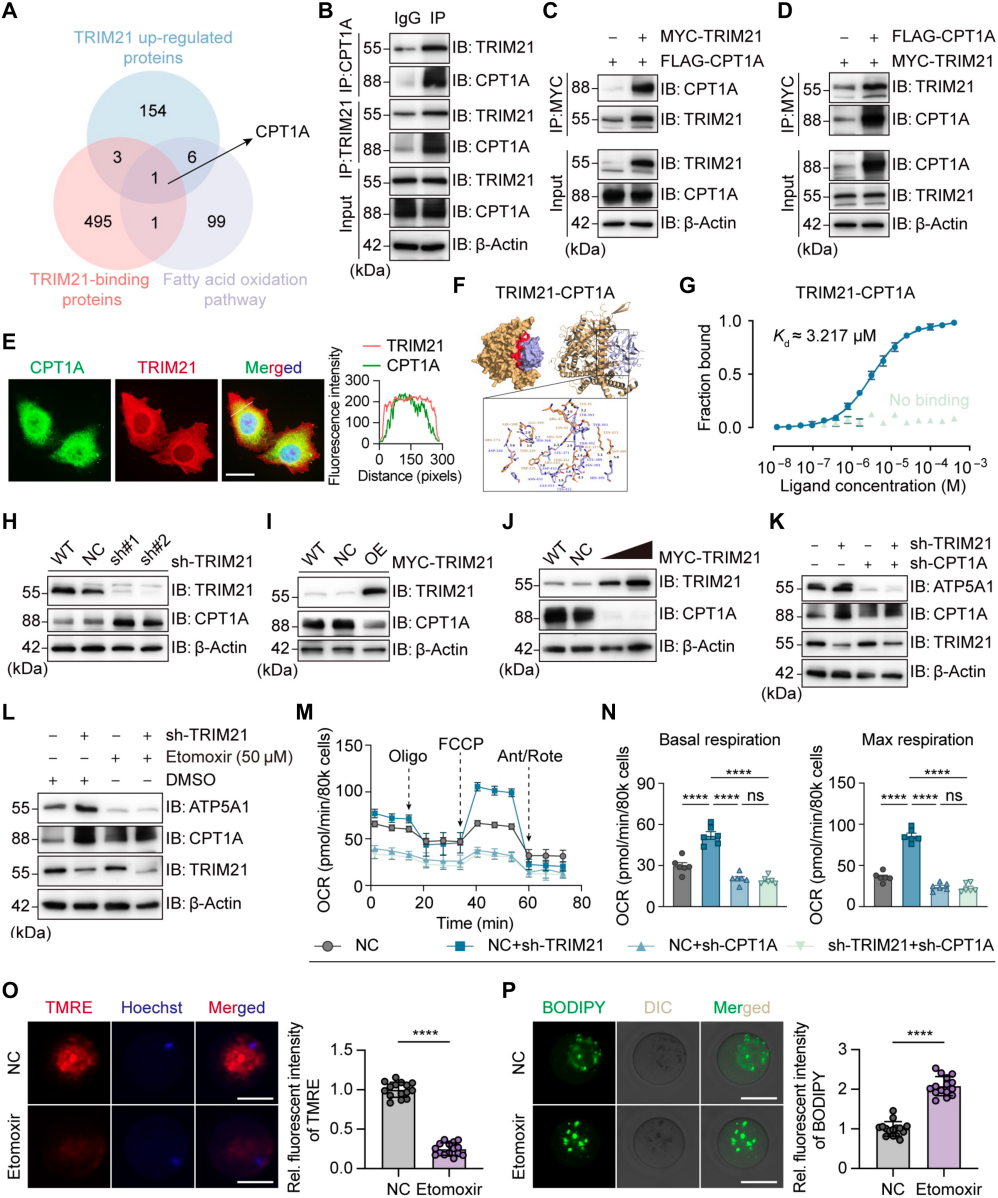
Tripartite motif-containing protein 21 (TRIM21) regulates fatty acid oxidation in granulosa cells through carnitine palmitoyltransferase 1A (CPT1A). (A) CPT1A was identified as a protein with high confidence interacting with TRIM21 by immunoprecipitation-mass spectrometry (IP-MS) and proteomic sequencing. (B) Western blot (WB) analysis of KGN cell lysates immunoprecipitated with TRIM21 or CPT1A antibodies. (C and D) Coimmunoprecipitation (Co-IP) and WB analysis of exogenous TRIM21 and CPT1A in KGN cells overexpressing Myc-TRIM21 and Flag- CPT1A. (E) Immunofluorescence staining ofTRIM21 and CPT1A by confocal microscopy (scale bar, 10 μm) in KGN cells. (F) Molecular docking of TRIM21 and CPT1A. (G) Microscale thermophoresis of TRIM21 and CPT1A. (H to J) WB analysis of CPT1A with TRIM21 silencing or overexpression in KGN cells. (K) WB analysis of ATP5A1 with CPT1A silencing, with or without TRIM21 knockdown in KGN cells. (L) WB analysis of ATP5A1 in cells with etomoxir, with or without TRIM21 knockdown in KGN cells. (M and N) Evaluation of the mitochondrial oxidative phosphorylation (OXPHOS) function by oxygen consumption rate (OCR), including basal respiration and maximum respiration (*n* = 6). (O) Representative images for tetramethylrhodamine ethyl ester perchlorate (TMRE) in oocytes (*n* = 15; scale bar, 50 μm) and relative fluorescence intensities. Three fields of view of each mouse were selected. (P) Representative images for lipid content in oocytes by (*n* = 15; scale bar, 50 μm) and relative fluorescence intensities. Three fields of view of each mouse were selected. Data are expressed as means ± standard error of the mean (SEM). ns, not significant; ****P < 0.0001. IB, immunoblot.

To identify the direct targets of TRIM21 in regulating FAO, we used immunoprecipitation-mass spectrometry (IP-MS) to detect proteins that bind to the TRIM21 protein. We then take the intersection of the following 2 datasets: The proteins belong to the top 500 proteins in the IP-MS results (Table S4), and the proteins were significantly up-regulated in the proteomics analysis of sh-TRIM21 cells (Fig. 2A). Also, the candidate protein is involved in FAO. Carnitine palmitoyltransferase 1A (CPT1A), a protein that is regulated by TRIM21 binding and participates in FAO, was present in the sets of results (Fig. 3A). CPT1A, a mitochondrial outer membrane protein, facilitates long-chain fatty acid import for β-oxidation. It is a key enzyme that regulates FAO and mitochondrial homeostasis [28]. We performed coimmunoprecipitation (Co-IP) in KGN cells to verify the endogenous binding of TRIM21 and CPT1A (Fig. 3B and Fig. S4A). Experiments using epitope-tagged proteins were performed in KGN cells, and Myc-TRIM21 and Flag-CPT1A coprecipitated in KGN cells (Fig. 3C and D). Immunofluorescence analysis revealed the colocalization of TRIM21 and CPT1A (Fig. 3E). Molecular docking experiments showed that TRIM21 can bind to CPT1A (Fig. 3F). Furthermore, Kd ≈ 3.217 μM has indicated the binding of TRIM21 and CPT1A through microscale thermophoresis (Fig. 3G). To verify the regulatory effect of TRIM21 on CPT1A, we knocked down and overexpressed TRIM21 in KGN and COV cells, respectively. Western blot analysis showed that TRIM21 knockdown led to increased CPT1A protein expression (Fig. 3H and Fig. S4I), while TRIM21 overexpression led to decreased CPT1A protein levels (Fig. 3I and Fig. S4C). Overexpression enhanced the regulatory effect of TRIM21 on CPT1A, leading to a further decrease in CPT1A expression (Fig. 3J). To test whether TRIM21 regulates ATP5A1 through CPT1A, we knocked down CPT1A and found that there was no difference in ATP5A1, regardless of whether TRIM21 was knocked down (Fig. 3K). The same results were obtained using the CPT1A inhibitor etomoxir (Fig. 3L). These results indicate that CPT1A is an indispensable target of TRIM21 in this process. Bioenergetic features were then investigated with the Seahorse XF on these GCs with different treatment. As shown in Fig. 3M and N, in sh-CPT1A GCs, there was no difference in the basal and maximum respiratory values, but they were significantly lower than the NC-CPT1A group, regardless of whether TRIM21 was knocked down, indicating that TRIM21 regulates mitochondrial function through CPT1A.

Next, we verified the results in the oocytes of mice. When etomoxir was added to the in vitro culture medium of COCs, the mitochondrial membrane potential of oocytes decreased (Fig. 3O), and lipid droplet aggregation occurred (Fig. 3P). RT-PCR was performed on key genes involved in oocyte development, and the mRNA levels of *Areg*, *Bmp15*, *Ereg*, and *Zp3* were significantly down-regulated in COCs exposed to etomoxir (Fig. S4 D to K). This shows that CPT1A has a direct effect on the FAO function of oocytes and follicular development. In summary, TRIM21 and CPT1A have a direct interaction and regulate FAO and mitochondrial OXPHOS in GCs through CPT1A, thereby affecting the development of oocytes.

### TRIM21 facilitates ubiquitination of CPT1A at lysine 161

**Fig. 4.**
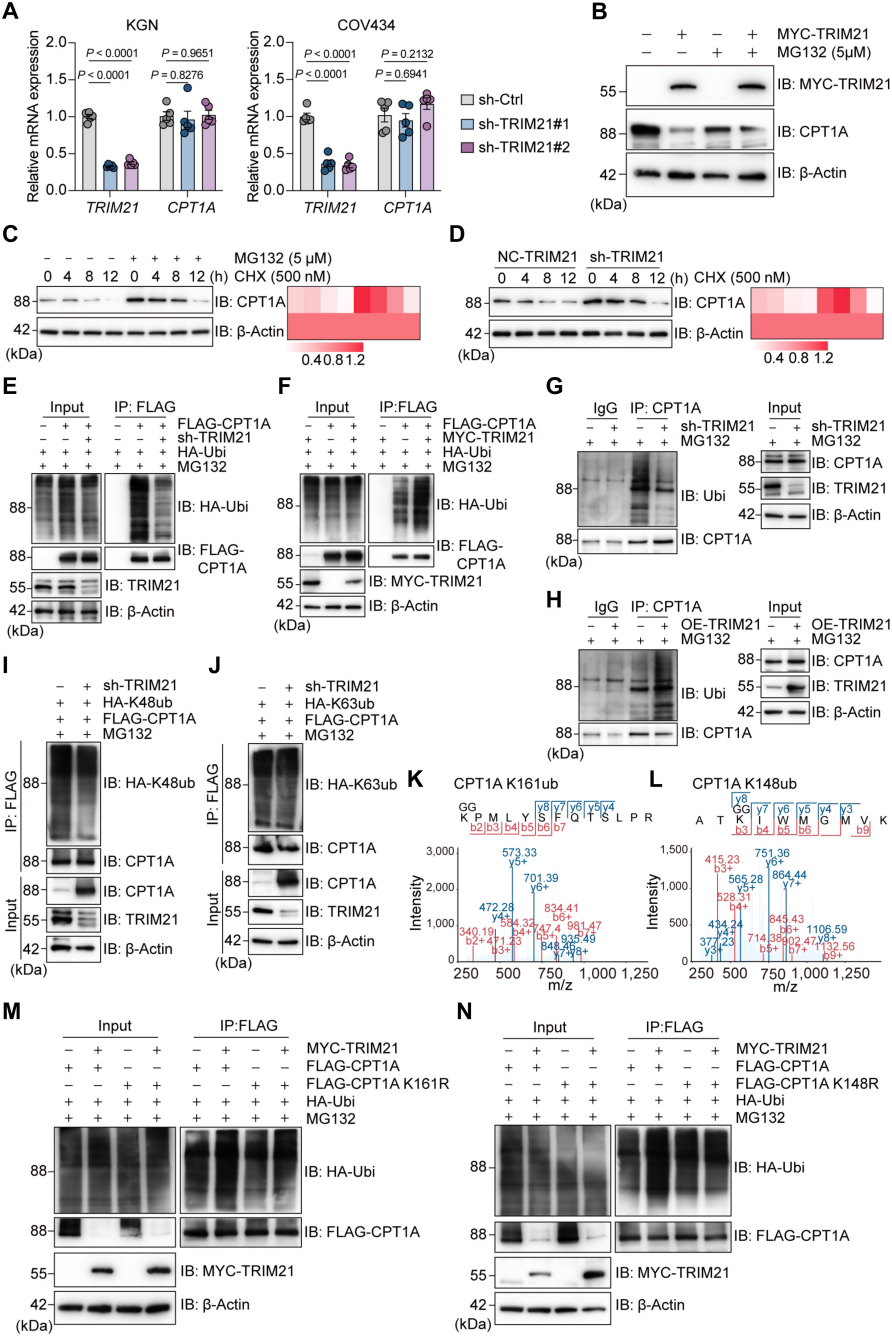
Tripartite motif-containing protein 21 (TRIM21) facilitates ubiquitination of carnitine palmitoyltransferase 1A (CPT1A) at lysine 161. (A) Reverse transcription polymerase chain reaction (RT-PCR) analysis of CPT1A with or without TRIM21 knockdown of KGN and COV434 cells (*n* = 5). (B) Western blot (WB) analysis of CPT1A with TRIM21 overexpression in the presence of MG132 in KGN cells. (C) WB analysis of CPT1A in KGN cells treated with cycloheximide (CHX) and MG132 at different time points. (D) WB analysis of CPT1A in cells with or without TRIM21 silencing in CHX-chase experiment in KGN cells. (E and F) In KGN cells, coimmunoprecipitation (Co-IP) and WB analysis of CPT1A exogenous ubiquitination with TRIM21 knockdown or overexpression in the presence of MG132 (10 μM, 4 h). (G and H) In KGN cells, Co-IP and WB analysis of CPT1A endogenous ubiquitination with TRIM21 knockdown or overexpression in the presence of MG132 (10 μM, 4 h). (I and J) In KGN cells, Co-IP and WB analysis of exogenous ubiquitination of CPT1A treated with Flag-CPT1A and HA-UB (wild type [WT], K48-only, and K63-only) in the presence of MG132 (10 μM, 4 h). (K and L) Analysis of ubiquitination modification sites of CPT1A by immunoprecipitation-mass spectrometry (IP-MS). (M and N) In KGN cells, Co-IP and WB of exogenous ubiquitination of CPT1A treated with Flag-CPT1A mutant plasmids in the presence of MG132 (10 μM, 4 h). Data are expressed as means ± SD. IB, immunoblot.

Given that TRIM21 is an E3 ubiquitin ligase and interacts with CPT1A, we tested whether TRIM21 down-regulates CPT1A protein levels in cells through ubiquitination. The relative expression of *CPT1A* mRNA after TRIM21 knockdown was measured using RT-PCR but showed no significant differences (Fig. 4A). Overexpression of TRIM21 in KGN cells reduced CPT1A protein levels, and this phenotype could be reversed by MG132 (a proteasome inhibitor) but not by the lysosome inhibitor bafilomycin A1 (Fig. 4B and Fig. S5A). MG132 also slowed down CPT1A degradation in the presence of the protein synthesis inhibitor cycloheximide (CHX; Fig. 4C). To test whether TRIM21 mediates the degradation of CPT1A, we conducted a CHX-chase experiment. Knocking down TRIM21 significantly delayed the degradation of the CPT1A (Fig. 4D). These results indicate that TRIM21 regulates the expression of CPT1A through a ubiquitin–proteasome system. To more intuitively study the effect of TRIM21 on CPT1A ubiquitination, we conducted an exogenous Co-IP experiment. Knocking down TRIM21 in KGN cells significantly reduced the ubiquitination level of CPT1A (Fig. 4E), while enhancing TRIM21 expression restored the ubiquitination of CPT1A (Fig. 4F). Next, the endogenous ubiquitination modification level of CPT1A was detected by ubiquitination immunoprecipitation experiments in KGN lines with knocked-down and overexpressed TRIM21, and consistent results were obtained (Fig. 4G and H). To explore the specific ubiquitination mode of TRIM21-mediated degradation of CPT1A, we used a group of ubiquitin mutants that specifically form K48 or K63 polyubiquitin linkages in ubiquitination experiments. Co-IP experiments based on exogenous ubiquitination showed that knockdown of TRIM21 could reduce the K48-linked polyubiquitination of the CPT1A protein but had no effect on K63-linked polyubiquitination (Fig. 4I and J). In summary, TRIM21 degrades CPT1A through K48-linked polyubiquitination.

We further studied the ubiquitination modification sites of CPT1A. CPT1A and an empty control plasmid were transfected into KGN cells, and exogenously expressed CPT1A was enriched by immunoprecipitation. After ubiquitination modification mass spectrometry analysis, 2 specific ubiquitination modification sites on the CPT1A protein were identified (Fig. 4K and L). These 2 lysines were mutated into arginine to construct a plasmid with inactivated ubiquitination modification sites. The wild-type and mutant plasmids of CPT1A were transfected and detected the polyubiquitination change in KGN cells. TRIM21 overexpression increased polyubiquitination of wild-type CPT1A but not the CPT1A with K161 mutant (Fig. 4M). Additionally, we found that the polyubiquitination of CPT1A with K148 mutant was consistent with that of the wild type (Fig. 4N). To verify whether the K161R mutation could restore the enzyme activity and cellular FAO flux of CPT1A, we performed functional complementation experiments on wild-type and K161 mutant CPT1A. We found that the K161 mutation did not definitely alter the ubiquitination level of CPT1A, which also resulted in no corresponding changes in CPT1A itself or its downstream protein ATP5A1 (Fig. S5B). Seahorse XF analysis revealed no significant difference in basal and maximum respiratory values between the K161 mutant CPT1A and the wild type (Fig. S5C and D). This result indicates that the K161 site of the CPT1A protein is a key site in the TRIM21-mediated ubiquitination of CPT1A.

### UBE2M promotes TRIM21 neddylation and its interaction with CPT1A

**Fig. 5.**
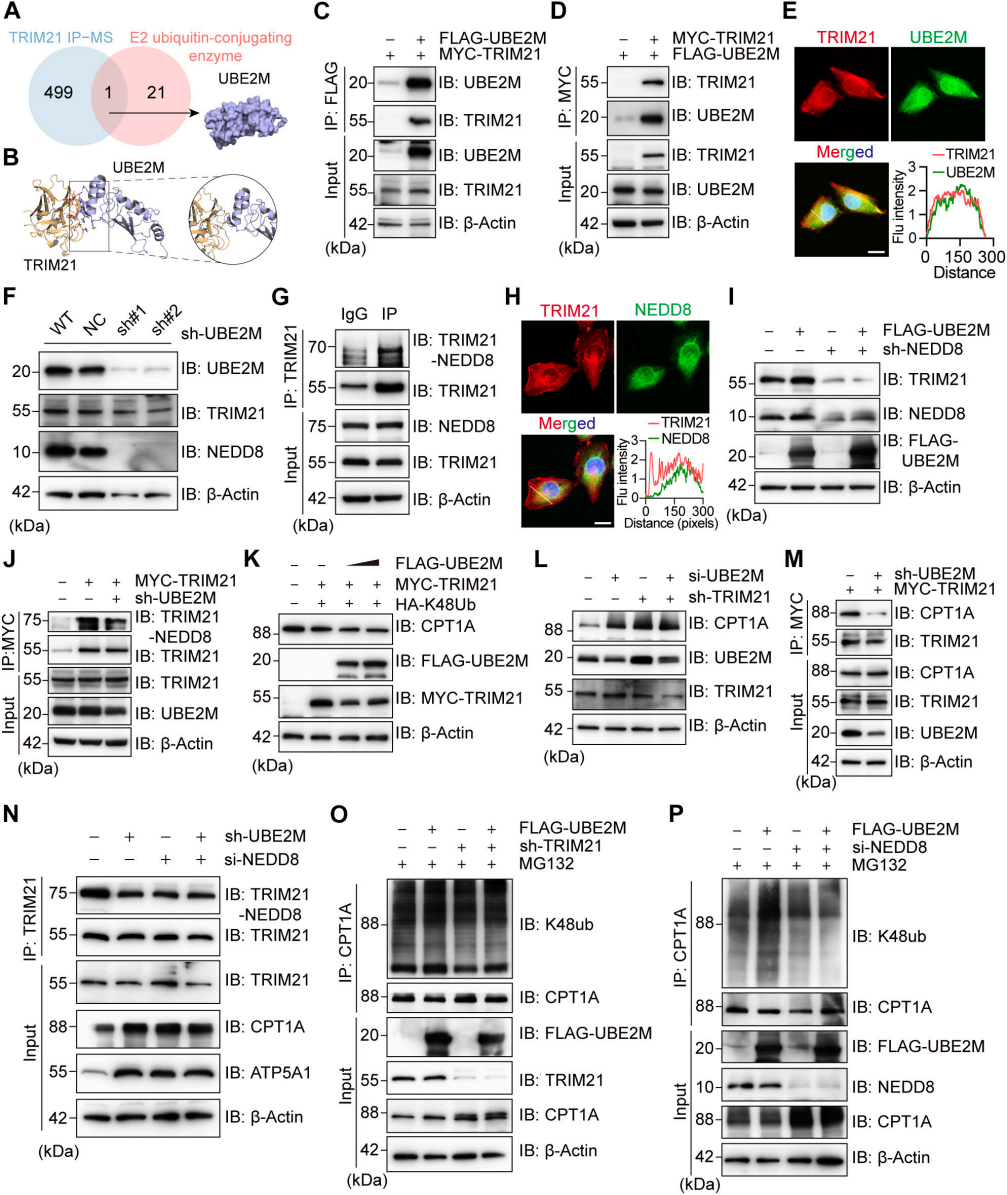
UBE2M promotes tripartite motif-containing protein 21 (TRIM21) neddylation and its interaction with carnitine palmitoyltransferase 1A (CPT1A). (A) UBE2M was identified as a protein with high confidence interacting with TRIM21 by immunoprecipitation-mass spectrometry (IP-MS) and functional analysis. (B) Molecular docking of UBE2M and TRIM21. (C and D) Coimmunoprecipitation (Co-IP) and Western blot (WB) analysis of exogenous UBE2M and TRIM21 in KGN cells overexpressing Flag-UBE2M and MYC-TRIM21. (E) Immunofluorescence staining of UBE2M and TRIM21 by confocal microscopy in KGN cells (scale bar, 20 μm). (F) WB analysis of TRIM21 and neural precursor cell-expressed developmentally down-regulated 8 (NEDD8) with UBE2M knockdown in KGN cells. (G) WB analysis of KGN cell lysates immunoprecipitated with immunoglobulin G (IgG) and anti-NEDD8 antibodies. (H) Immunofluorescence staining of TRIM21 and NEDD8 by confocal microscopy in KGN cells (scale bar, 20 μm). (I) WB analysis of TRIM21 in KGN cells overexpressing UBE2M with or without NEDD8 knockdown. (J) Co-IP and WB analysis of TRIM21 neddylation with or without UBE2M knockdown in KGN cells. (K) WB analysis of CPT1A in KGN cells treated with different concentrations of FLAG-UBE2M. (L) WB analysis of CPT1A treated with UBE2M silencing, with or without TRIM21 knockdown in KGN cells. (M) Co-IP and WB analysis of KGN cell lysates immunoprecipitated with anti-MYC antibody in the presence of UBE2M knockdown. (N) In KGN cells, Co-IP and WB analysis of TRIM21 neddylation, CPT1A and ATP5A1 with UBE2M silencing, with or without NEDD8 knockdown. (O) In KGN cells, WB analysis of K48 ubiquitination of CPT1A in cells with UBE2M overexpression, with or without TRIM21 knockdown in the presence of MG132 (10 μM, 4 h) treatment. (P) In KGN cells, Co-IP and WB analysis of exogenous K48 ubiquitination of CPT1A in KGN cells with UBE2M overexpression, with or without NEDD8 knockdown in the presence of MG132 (10 μM, 4 h). IB, immunoblot.

As a ubiquitin ligase, TRIM21 needs to form a complex with a specific ubiquitin-conjugating enzyme (E2) in the ubiquitination cascade system to ubiquitinate and degrade the substrate. The candidate proteins should both bind to TRIM21 based on the IP-MS (Table S4) and belong to ubiquitin-conjugating enzymes (Table S5). According to the above, we found that ubiquitin conjugating enzyme E2 M (UBE2M) and TRIM21 have strong binding and interaction potential (Fig. 5A). Molecular docking predicted that UBE2M and TRIM21 can bind (Fig. 5B). Subsequently, we confirmed the binding of UBE2M and TRIM21 in KGN cells through exogenous and endogenous Co-IP (Fig. 5C and D and Fig. S5E and F). Immunofluorescence by confocal microscopy showed that UBE2M and TRIM21 colocalized in KGN cells (Fig. 5E). Next, we investigated how UBE2M interacts with TRIM21. After knocking down UBE2M in KGN cells, the amount of TRIM21 protein decreased (Fig. 5F). UBE2M participates in the neddylation pathway by mediating the transfer of the small neural precursor cell-expressed developmentally down-regulated 8 (NEDD8) protein to target proteins [29,30]. We assessed whether UBE2M in PCOS can regulate protein level of TRIM21 by targeting neddylation. NEDD8 expression was reduced after knocking down UBE2M in KGN cells (Fig. 5F), and NEDD8 bands were detected in immunoblotting of endogenous TRIM21 immunoprecipitation (Fig. 5G). Immunofluorescence also suggested that TRIM21 and NEDD8 colocalized (Fig. 5H). The expression of NEDD8 protein was down-regulated after knocking down TRIM21 in KGN cells (Fig. S5D), proving that TRIM21 binds to NEDD8. Interestingly, we confirmed that the UBE2M overexpression-mediated increases of TRIM21 in KGN cells were abolished after NEDD8 knockdown (Fig. 5I), indicating that UBE2M regulates TRIM21 through NEDD8. We next studied the regulation of TRIM21 neddylation by UBE2M and found that the level of endogenous TRIM21 neddylation decreased after knocking down UBE2M in KGN cells (Fig. 5J), while the level of endogenous TRIM21 neddylation increased after overexpression of UBE2M (Fig. S5E).

To determine whether UBE2M also regulates CPT1A, we overexpressed UBE2M in KGN cells and found that CPT1A was decreased (Fig. 5K). Next, we knocked down and overexpressed UBE2M and found that the difference in CPT1A expression disappeared when TRIM21 was knocked down (Fig. 5L and Fig. S5F). Therefore, we propose that UBE2M mediates the change in CPT1A through TRIM21. In knockdown UBE2M KGN cells, the interaction between exogenous TRIM21 and CPT1A was reduced (Fig. 5M). In contrast, overexpression of UBE2M enhanced the interaction (Fig. S5G). Therefore, UBE2M enhances the interaction between TRIM21 and CPT1A. Next, to determine whether UBE2M causes changes in CPT1A via neddylation of TRIM21, we silenced UBE2M and NEDD8 in KGN cells, respectively, to evaluate the expression of CPT1A and ATP5A1. Interestingly, NEDD8 silencing reduced the neddylation of TRIM21. Because of the decreased expression of TRIM21, the expression of CPT1A and ATP5A1 increased (Fig. 5N). To determine whether UBE2M affects the E3 ubiquitin ligase activity of TRIM21, we overexpressed UBE2M in KGN cells and found that the ubiquitination of CPT1A increased, but its polyubiquitination was eliminated by TRIM21 knockdown (Fig. 5O). In addition, we obtained the same result when knocking down NEDD8 (Fig. 5P). Therefore, we propose that UBE2M modifies TRIM21 through neddylation and thus affects its activity as an E3 ubiquitin-conjugating enzyme.

### MLN4924 reverses the ubiquitination of CPT1A by TRIM21 and ameliorates the phenotype of PCOS mice

**Fig. 6.**
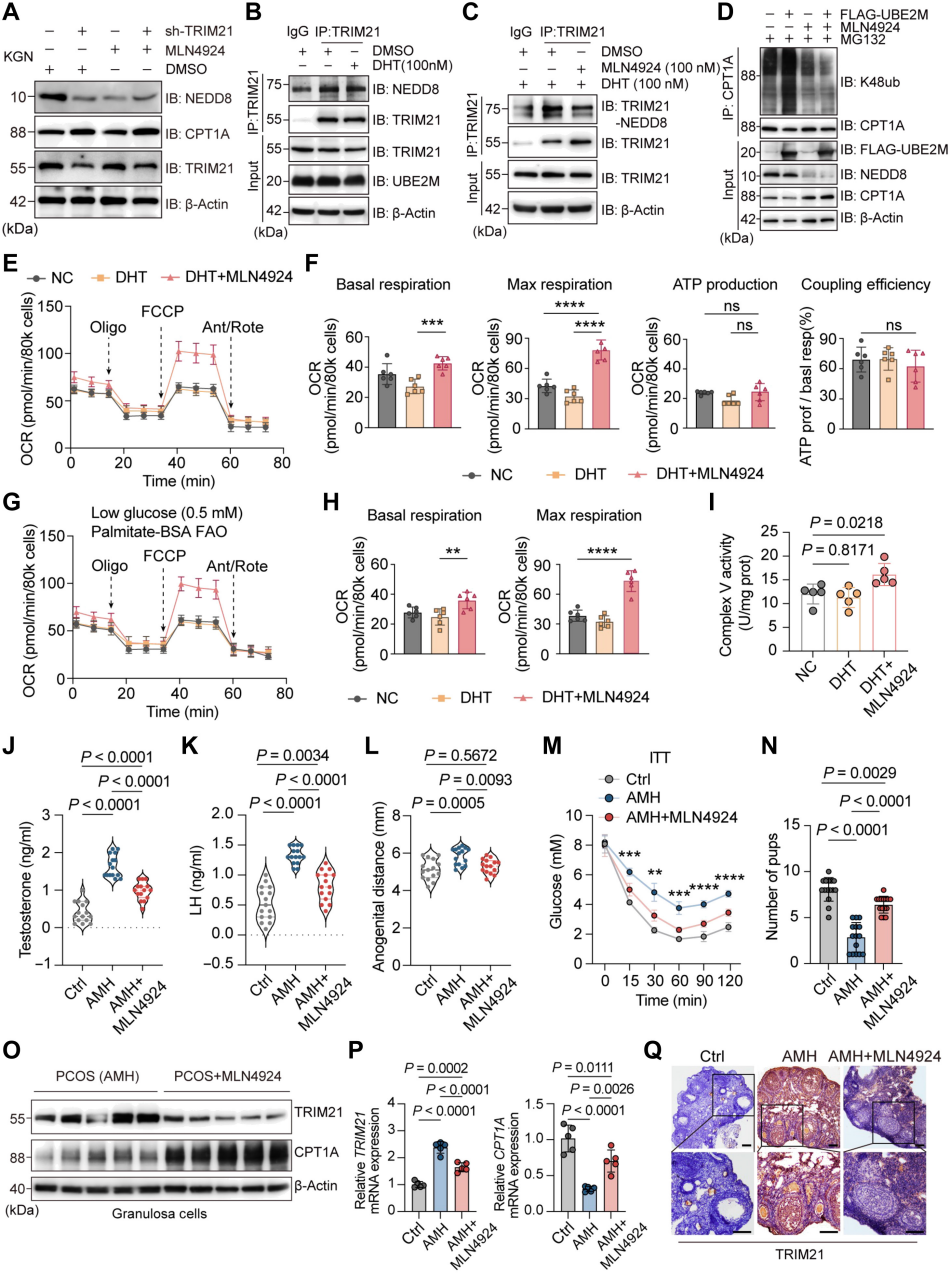
MLN4924 reverses the ubiquitination of carnitine palmitoyltransferase 1A (CPT1A) by tripartite motif-containing protein 21 (TRIM21) and ameliorates the phenotype of polycystic ovary syndrome (PCOS) mice. (A) Western blot (WB) analysis of neural precursor cell-expressed developmentally down-regulated 8 (NEDD8) and CPT1A treated with MLN4924, with or without TRIM21 knockdown in KGN cell. (B and C) Coimmunoprecipitation (Co-IP) and WB analysis of TRIM21 neddylation in KGN cells treated with or without MLN4924. (D) Co-IP and WB analysis of exogenous K48 ubiquitination of CPT1A in KGN cells overexpressing UBE2M, with or without NEDD8 knockdown in the presence of MG132 (10 μM, 4 h). (E and F) Evaluation of the mitochondrial oxidative phosphorylation (OXPHOS) function by oxygen consumption rate (OCR) in KGN cells, including basal respiration, maximum respiration, ATP generation, and coupling efficiency (*n* = 6). (G and H) Evaluation of fatty acid oxidation (FAO)-dependent mitochondrial function by OCR in KGN cells, including basal respiration and maximum respiration (*n* = 6). (I) Activity of mitochondrial complex V in KGN cells (*n* = 6). (J and K) Testosterone and luteinizing hormone (LH) levels of serum (*n* = 15). (L) Anogenital distance in adult female mice (*n* = 15). (M) Insulin tolerance test (ITT) test in adult female mice after 4 h of fasting (*n* = 5). (N) Number of pups per birth (*n* = 10). (O and P) WB and reverse transcription polymerase chain reaction (RT-PCR) analysis of TRIM21 and CPT1A in ovarian granulosa cells (GCs). (Q) Immunohistochemical staining of TRIM21 in mouse ovarian tissues (scale bars, 100 μm). Data are expressed as means ± standard error of the mean (SEM), and each symbol represents a biologically independent mouse. Significance was calculated by 1-way analysis of variance (ANOVA) multiple comparison test. Blood glucose analysis between groups (M) was determined by 2-way ANOVA and multiple comparison test. ns, not significant; **P < 0.01; ***P < 0.001; ****P < 0.0001. IB, immunoblot.

Previous studies have found that inhibiting the neddylation of TRIM21 can reduce its protein expression. MLN4924 (pevonedistat) is a small-molecule inhibitor that acts on the NEDD8-activating enzyme and inhibits neddylation [31]. To determine whether MLN4924 could reverse TRIM21-mediated CPT1A ubiquitination and degradation, we added MLN4924 and found that CPT1A expression increased with down-regulation of NEDD8 and TRIM21 expression, and this increase disappeared in knockdown TRIM21 KGN cells (Fig. 6A). Meanwhile, we added different doses of MLN4924 and found that CPT1A increased with the down-regulation of NEDD8 and TRIM21 (Fig. S5K). Next, DHT was added to KGN cells, and the degree of TRIM21 neddylation increased (Fig. 6B), but this effect decreased after the addition of MLN4924 (Fig. 6C). In KGN cells overexpressing UBE2M, the ubiquitination level of CPT1A was down-regulated after the addition of MLN4924, but its polyubiquitination was eliminated by TRIM21 knockdown (Fig. 6D), indicating that MLN4924 can inhibit the neddylation of TRIM21 while increasing the amount of CPT1A protein. The bioenergetics characteristics of KGN cells after the addition of MLN4924 were studied using Seahorse XF analysis. As shown in Fig. 6E and F, the basal respiration and maximum respiration of cells were up-regulated after the addition of MLN4924, while there was no significant difference in ATP generation and coupling efficiency between groups. In addition, both the baseline and the capacity of glycolysis showed a downward trend with the addition of MLN4924 (Fig. S6A and B). Noting that MLN4924 is also an antitumor drug, a decrease in the uptake and utilization of glucose by cells is likely. The maximum respiration value dependent on FAO increased apparently after the addition of MLN4924 (Fig. 6G and H), suggesting that MLN4924 improved mitochondrial function related to FAO in ovarian GCs. The expression of respiratory chain complex V increased after the addition of MLN4924 to KGN cells (Fig. 6I and Fig. S6C), which is consistent with the results of knocking down TRIM21. In summary, MLN4924 can reverse the ubiquitination-mediated degradation of CPT1A by TRIM21, while improving mitochondrial respiration function dependent on FAO.

To determine whether MLN4924 can improve the phenotype of PCOS mice, we treated a PCOS mouse model based on AMH and DHT with MLN4924. MLN4924 reduced the serum testosterone and luteinizing hormone of PAMH F_1_ mice (Fig. 6J and K), restored the normal estrous cycle (Fig. S6D), normalized the AGD (Fig. 6L), and showed a trend toward improved insulin sensitivity (Fig. 6M), although there was no difference in glucose tolerance (Fig. S6E). The same phenotypic changes were seen in DHT mice (Fig. S6F to J), except that the weight of mice in the DHT group was restored after treatment (Fig. S7A). Importantly, MLN4924 improved the fertility of PCOS mice, as shown by an increase in the number of pups per litter after treatment (Fig. 6N and Fig. S7B). To determine the mechanism of action of MLN4924, we performed western blotting and RT-PCR on mouse ovarian GCs. CPT1A expression was increased, and TRIM21 expression was down-regulated in the MLN4924-treated mice (Fig. 6O and P and Fig. S7C and D), and the same results were obtained by immunohistochemistry of ovarian tissue (Fig. 6Q and Fig. S7E). Overall, MLN4924 treatment improved the phenotype of PCOS in the mouse model, including impaired glucose tolerance, increased serum testosterone, irregular estrous cycle, and subfertility.

### MLN4924 improves the quality of oocytes of PCOS mice

**Fig. 7.**
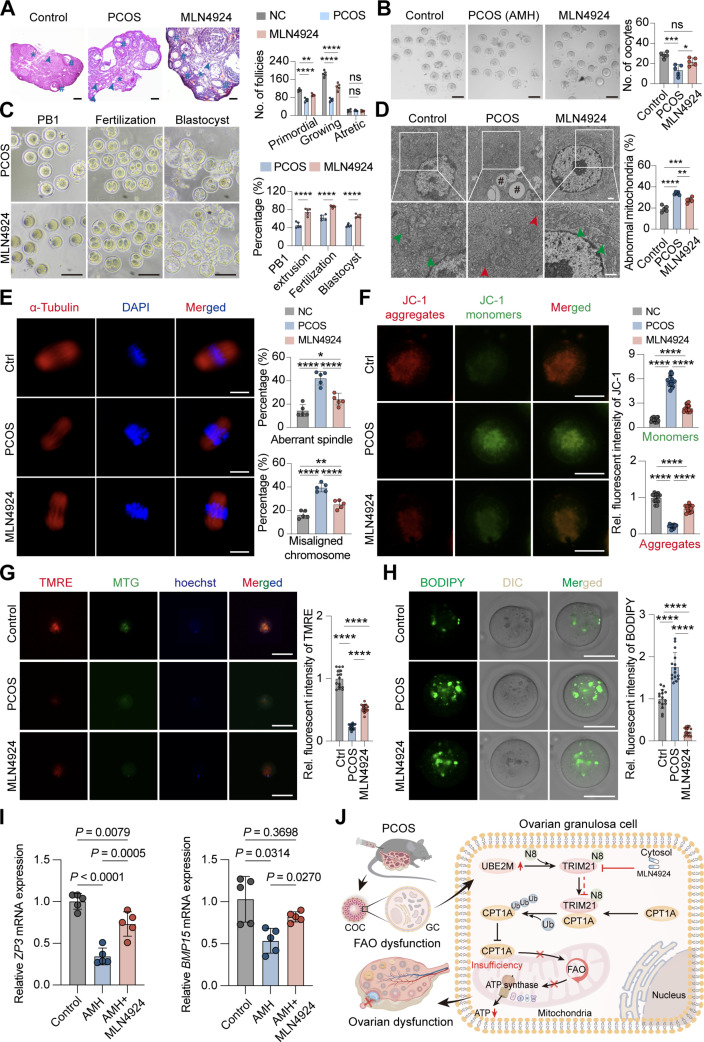
MLN4924 improves the quality of oocytes of polycystic ovary syndrome (PCOS) mice. (A) Hematoxylin and eosin (H&E) staining of ovaries and the number of follicles in ovary including primordial follicles (*), growing follicles (#), and atretic follicles (arrows; scale bars, 100 μm). (B) Number of oocytes after superovulation (*n* = 5 mice; scale bars, 100 μm). (C) Representative images and percentage of first polar body extrusion in mouse oocytes, fertilization rate, and blastocyst maturation rate (*n* = 5 mice; scale bars, 100 μm). (D) Transmission electron microscopy (TEM) analysis of mitochondrial morphology of mouse ovarian granulosa cells. Green arrows indicate normal mitochondria, red arrows indicate abnormal mitochondria, and # indicates lipid droplets (*n* = 5 mice; scale bar, 500 nm). (E) Representative images of spindle morphology and chromosome alignment in oocytes at the metaphase II stage by confocal microscopy (*n* = 5 mice; scale bar, 10 μm). The percentage of aberrant spindles and misaligned chromosomes were quantified in oocytes at metaphase II from control (*n* = 23), PCOS (*n* = 19), and MLN4924 (*n* = 21) mice. (F) Representative images for JC-1 staining of mouse oocytes (*n* = 15; scale bar, 50 μm) and relative fluorescence intensities. Three fields of view of each mouse were selected (G) Representative images for tetramethylrhodamine ethyl ester perchlorate (TMRE) staining of mouse oocytes (*n* = 15; scale bar, 50 μm) and relative fluorescence intensities. Three fields of view of each mouse were selected. (H) Representative images for lipid content in oocytes (*n* = 15; scale bar, 50 μm) and ratio of the fluorescence intensities. Three fields of view of each mouse were selected. (I) Reverse transcription polymerase chain reaction (RT-PCR) analysis of mRNA levels of follicle development-related genes in mouse cumulus–oocyte complexes (COCs) (*n* = 5). Data are expressed as means ± SD, and each symbol represents a biologically independent mouse. (J) Schematic diagram of the pathway by which tripartite motif-containing protein 21 (TRIM21) regulates ubiquitination of carnitine palmitoyltransferase 1A (CPT1A) leading to abnormal fatty acid oxidation in ovarian granulosa cells. ns, not significant; *P < 0.05; **P < 0.01; ***P < 0.001; ****P < 0.0001.

Abnormal follicular development and decreased fertility are 2 of the most prominent problems of PCOS. To study whether MLN4924 improves the quality of follicles while improving the PCOS phenotype in mice, we histologically analyzed the ovaries of mice and found that the total number of primordial follicles and growing follicles increased after MLN4924 treatment, while atretic follicles show no difference (Fig. 7A).

The oocytes obtained by superovulation of mice were counted and morphologically evaluated. MLN4924 treatment elevated oocyte yield (Fig. 7B), which means that oligo-ovulation in the AMH group was improved. In the in vitro maturation (IVM) of oocytes experiment, the first polar body extrusion rate of oocytes in the treatment group increased (Fig. 7C), indicating that the maturation ability of oocytes was improved. Confocal imaging showed that the incidence of spindle abnormalities and chromosome misalignment in oocytes in the treatment group was significantly reduced compared with PCOS mice (Fig. 7E), further indicating that the meiotic process was improved. The in vitro fertilization results showed that the 2-cell formation rate and blastocyst formation rate increased after treatment (Fig. 7C), indicating that MLN4924 improved the embryonic development potential of PCOS mice. IVM and early embryonic development are highly dependent on mitochondrial energy production. We used TEM to observe the ultrastructure of mouse ovarian GCs. PCOS mice had fewer mitochondria, and some were vacuolated, with increased lipid droplets accompanied by mitochondrial crista breakage. After MLN4924 treatment, the number of mitochondria increased, and the cristae were evenly distributed (Fig. 7D). Next, confocal microscopy analysis of oocyte mitochondrial function showed that MLN4924 improved mitochondrial membrane potential in PCOS mice through JC-1 and TMRE (Fig. 7F and G). In addition, the neutral lipid content of oocytes in the MLN4924 group was decreased compared with the PCOS group (Fig. 7H), indicating increased utilization of fatty tissue. Next, we used RT-PCR to evaluate the oocyte quality and developmental markers after MLN4924 treatment. The mRNA levels of *Bmp15* and *Zp3* in the treatment group were significantly increased compared with the PCOS group (Fig. 7I and Fig. S7F). This indicates that MLN4924 can improve the follicle quality and oocyte development ability of PCOS mice by improving mitochondrial function and FAO.

## Discussion

In the present study, we found elevated TRIM21 protein expression in the PAMH F_1_ mouse model, and similar results were obtained in the DHT mouse model. Analysis of TRIM21 indicated that it was closely related to FAO and mitochondrial function in ovarian GCs through the regulation of the key enzyme CPT1A for fatty acid β-oxidation via ubiquitination at the K161 site leading to degradation of CPT1A. We also found that TRIM21, an E3 ubiquitin ligase, was regulated by the ubiquitin-conjugating enzymes UBE2M, and UBE2M-mediated neddylation contributed to the regulation of TRIM21 protein levels. The TRIM21 neddylation inhibitor MLN4924 improved FAO and mitochondrial function in GCs. MLN4924 also partially alleviated the phenotype of PCOS, including sex hormone disorders and insulin resistance, and improved the quality of oocytes and embryonic development potential (Fig. 7J).

In view of the common pathological characteristics of hyperandrogenism and ovarian dysfunction in PCOS patients, we used DHT and PAMH mouse models to construct 2 PCOS mouse models. As a nonaromatizable androgen, exogenous supplementation of DHT can accurately simulate the hyperandrogenism of PCOS patients. By interfering with the hormone balance in the body, it effectively inhibits the normal maturation and ovulation of follicles, thereby inducing secondary polycystic changes in the ovary [32]. The PAMH mouse model simulates the effects of high maternal AMH levels on the fetus during human embryonic development by exposing pregnant mice to elevated AMH levels. It is closer than DHT model to the natural PCOS pathogenesis in humans and has neuro-endocrine-metabolic integration [33–35]. Through the 2 PCOS mouse models, we successfully reproduced the pathological characteristics of PCOS patients in mice from the perspectives of reproductive endocrine and metabolic abnormalities and reproductive development disorders, thus providing strong support for our research results.

Anovulation caused by abnormal follicular development is the most critical feature of ovarian dysfunction in PCOS, yet the underlying mechanism remains unclear [36]. Previous studies have found that there are remarkable glycolysis pathway defects and cholesterol metabolism disorders in the ovarian GCs of PCOS patients [37,38]. The glycolysis pathway in GCs is a key pathway for the energy supply of oocytes, and its metabolic-level defects are a direct factor leading to impaired oocyte development and reduced pregnancy rate in PCOS patients [39,40]. Impaired cholesterol synthesis in GCs can cause steroid hormone metabolism disorders and interfere with the normal oocyte development and maturation [12,41]. In this study, we showed that FAO in the ovarian GCs of PCOS mice was markedly impaired. FAO is a core part of lipid metabolism, which oxidizes fatty acids in mitochondria to ultimately generate ATP. The development of follicles from primordial follicles to ovulated mature follicles is an energy-intensive process, in which FAO plays a key role in energy support [6,7]. Mitochondrial dysfunction in patients with PCOS may be a factor leading to poor folliculogenesis. Studies have provided that adding moderate doses of fatty acids to IVM culture medium is beneficial to oocytes and blastocysts [42,43]. β-Aminoisobutyric acid promotes lipid catabolism by activating adenosine 5'-monophosphate-activated protein kinase and augments the developmental competence of oocytes [44]. In addition, improving mitochondrial dysfunction can reduce the proportion of abnormal spindle structures and chromosome arrangement in oocytes and improve their ability to fertilize and develop into blastocysts [45,46]. These findings provide strong supporting evidence for the dependence of oocytes on GC FAO and offer novel insights underlying the impaired fertility of PCOS patients. However, more experimental studies are required to analyze the intrinsic mechanisms involved in this process.

Through transcriptomics and proteomics analysis, the expression of TRIM21 in ovarian GCs of PCOS mice showed an up-regulated trend. We performed proteomic enrichment analysis on KGN cells with TRIM21 knockdown and found that the expression of genes related to FAO was significantly changed. In recent years, the regulation of metabolic homeostasis by TRIM21 has attracted attention. Studies have shown that TRIM21 ubiquitin degradation of γ-butyrobetaine dioxygenase, a key enzyme in de novo carnitine synthesis, induces metabolic dysfunction-associated steatotic liver disease [47], and inhibition of TRIM21 may decrease lipotoxicity caused by long-chain free fatty acids [48]. In this study, we identified CPT1A as a candidate binding protein for TRIM21 that regulates lipid metabolism. CPT1A, located on the mitochondrial outer membrane, shuttles acyl-coenzyme A via the carnitine system to participate in the energy supply of mitochondrial fatty acid β-oxidation. Therefore, carnitine deficiency will lead to insufficient energy generation, affect oocyte quality, and thus reduce fertility potential [49,50]. In this study, we demonstrated that TRIM21 is a ubiquitin ligase that promotes binding to K48-linked ubiquitin moieties at the CPT1A K161 site and directs the ubiquitin–proteasome system to degrade CPT1A, thereby substantially affecting the efficiency of fatty acids entering the mitochondria and participating in the oxidation process. These results support the view that abnormal TRIM21 function inhibits the FAO pathway and disrupts the energy production of ovarian GCs, positioning TRIM21 as a key pathogenic factor in PCOS ovarian dysfunction.

Our results also highlight the coordinated regulatory mechanism of 2 core protein posttranslational modifications, ubiquitination and neddylation, in the PCOS pathological model. TRIM21 is a ubiquitin ligase (E3), and its activity depends on specific ubiquitin-conjugating enzymes (E2), such as UBE2N and UBE2V1/V2 complexes [24,51]. Through IP-MS, we found that this process is regulated by UBE2M, a NEDD8-specific E2 conjugating enzyme. NEDD8 is a ubiquitin-like protein (ULP) with 80% homology to ubiquitin. NEDD8 is posttranslationally modified by covalently binding to protein lysine residues, a process named neddylation [52]. Further studies showed that UBE2M-mediated TRIM21 neddylation up-regulated the ubiquitination level of CPT1A, causing considerable defects in CPT1A and abnormal FAO function in GCs. Therefore, abnormal regulation of protein neddylation plays an upstream regulatory role in the pathological process of PCOS.

To evaluate the potential of disrupting TRIM21 as a treatment for PCOS, we first applied the small-molecule drug MLN4924 in a PCOS mouse model. As an inhibitor of NEDD8-activating enzyme, MLN4924 has attracted widespread attention for its wide range of applications including antilymphoblastic leukemias, alleviation of diet-induced hepatic steatosis, and endothelial dysfunction caused by atherosclerosis [53–55]. The application of MLN4924 inhibited TRIM21 neddylation, thereby improving the FAO of PCOS ovarian GCs and alleviating oocyte development disorders caused by abnormal FAO. Pharmacological blockade of TRIM21 neddylation in GCs by MLN4924 may be a promising therapeutic strategy for PCOS and PCOS-related ovulatory disorders.

However, several translational considerations should be carefully addressed. MLN4924 is a first-in-class inhibitor of the NEDD8-activating enzyme and broadly suppresses neddylation of cullin–RING ligases, which are involved in multiple cellular pathways beyond ovarian regulation [56,57] Therefore, systemic administration may pose risks related to off-target effects, reproductive toxicity, and long-term safety, particularly given the chronic nature of PCOS as a metabolic and endocrine disorder. Consistent with this concern, clinical studies of MLN4924 in oncology have reported hematologic and gastrointestinal adverse events, highlighting the limitations of directly repurposing this agent for nononcological indications [58,59]. To address these limitations, future studies should explore strategies to improve tissue specificity and reduce systemic exposure. Localized ovarian delivery approaches, such as intraovarian administration or sustained-release formulations, may represent potential options to enhance ovarian drug availability while limiting extraovarian effects. Although related strategies have been applied in other ovarian conditions and assisted reproductive contexts (via ultrasound-guided injection or sustained-release biomaterials), their applicability to PCOS requires rigorous evaluation [60,61]. Overall, our findings support the TRIM21-neddylation axis as a mechanistically relevant pathway in PCOS pathophysiology. However, further work is required to establish the feasibility, safety, and translational relevance of targeting this pathway before clinical application can be considered.

In summary, this study explored how TRIM21 in PCOS participates in FAO and affects follicular development by ubiquitinating and degrading CPT1A, deepening the understanding of GC lipid metabolism disorders in PCOS-related fertility defects. Based on the finding that TRIM21 ubiquitinates and degrades CPT1A and is itself regulated by neddylation, TRIM21 neddylation shows promise in the treatment of PCOS and may have clinical potential.

## Materials and Methods

### Mouse models

Four-week-old C57BL/6J mice (Beijing Huafukang Bio-Technology Co., Ltd.) were housed in specific-pathogen-free facilities (12-h light/12-h dark cycle, 22 ± 1 °C, 60% humidity) with ad libitum access to food and water. Two distinct PCOS models were established: The first involved PMAH (PAMH) therapy, where pregnant dams received daily intraperitoneal injections of AMH (Cloud-Clone Corp., cat# RPA228Hu01) at 0.12 mg/kg from gestational days 16.5 to 18.5, resulting in AMH F_1_ offspring; the second involved female mice subcutaneously injected with DHT (Glpbio, cat# GC19064) dissolved in sesame oil (Solarbio, cat# IS9030) at 6 mg/100 g body weight daily for 35 consecutive days. All animal procedures were approved by the Animal Care and Use Committee of Shengjing Hospital, China Medical University (Approval No. 2024PS001K) and were conducted in accordance with the National Institutes of Health guidelines for the care and use of laboratory animals.

### Estrous cycle staging

Estrous cycle determination was performed on adult mice aged 8 to 16 weeks for 21 consecutive days. Vaginal smears were collected daily at 8:00 AM by flushing the vagina with 10 μl of 0.9% sterile saline. The collected samples were then transferred onto glass slides, air-dried, and subsequently stained with hematoxylin and eosin for microscopic assessment based on vaginal cytology. Mice exhibiting all 4 estrous stages within a 4- to 6-d period were classified as regularly cycling.

### Serum hormone assessment

Circulating levels of testosterone (Fine test, cat# EM1850-HS) and luteinizing hormone (Fine test, cat# EM1188) were quantified in whole blood samples from mice using enzyme-linked immunosorbent assay kits. Mice were euthanized under isoflurane anesthesia, and cardiac exsanguination samples were collected simultaneously from all groups. Samples were centrifuged (2,800 × g, 10 min, 4 °C), and serum was preserved at −80 °C for subsequent experiments.

### Ovarian hematoxylin and eosin staining

Following phosphate-buffered saline (PBS) rinsing, ovaries were fixed in 4% paraformaldehyde at 4 °C for 48 h. Subsequently, fixed ovarian samples underwent dehydration and paraffin embedding. Blocks were then sectioned at 5 μm and deparaffinized for hematoxylin (Servicebio, cat# G1039-500ML) and eosin (Servicebio, cat# G1002-100ML) staining. Stained sections were dehydrated through ascending ethanol concentrations and coverslipped with neutral resin. All staining procedures adhered to the manufacturers’ instructions. Ovarian morphology and oocyte quality were characterized by quantifying the total number of primordial, growing, and atretic follicles using a microscope (Nikon, ci-L, JAPAN). Approximately 40 to 60 slides were observed per mouse for this assessment.

### Reproductive phenotyping

AGD refers to the midline from the center of the anus to the base of the external genitalia and was measured in adult mice using a vernier caliper. To minimize measurement error, each mouse was measured 3 times, and the average measurement was recorded. Fertility was assessed by observing the presence of a vaginal plug on the day after mating. Pregnancy was confirmed by examining implantation sites at day 10 postmating, following humane euthanasia. The remaining female mice delivered naturally, and first-litter pup numbers were counted.

### Insulin tolerance test and glucose tolerance test

For the insulin tolerance test, mice were fasted for 4 h (10:00 AM to 2:00 PM) prior to intraperitoneal injection of recombinant human insulin (0.75 IU/kg body weight). Blood glucose was subsequently monitored at 0, 15, 30, 60, 90, and 120 min postinsulin administration via tail vein bleeding with a glucometer (OMRON). For the glucose tolerance test, mice underwent a 16-h fast (5:00 PM to 09:00 AM) before intraperitoneal administration of d-glucose (2 mg/g body weight). Blood glucose was then recorded at 0, 15, 30, 45, 60, 90, and 120 min following administration, also by tail vein bleeding with a glucometer (OMRON).

### Primary mouse ovarian GC isolation and culture

Four-week-old C57BL/6J mice received an intraperitoneal injection of 20 IU of pregnant mare serum gonadotropin (Ningbo Second Hormone Factory). After 48 h, mice were sacrificed, and both ovaries were collected. Ovarian GCs were isolated by needle puncture, passed through a 40-μm cell sieve, and subsequently centrifuged at 1,000 rpm for 5 min. The isolated cells were thereafter resuspended in Dulbecco's modified Eagle medium/F12 medium (KeyGEN, cat# KGL1202-500) containing 10% fetal bovine serum (Vivacell, cat# C04001-500) and 1% streptomycin–penicillin (KeyGEN, cat# KGL2303-100) and cultured at 37 °C, 5% CO_2_. Immunofluorescence staining for the GC-specific marker, follicle-stimulating hormone receptor (Proteintech, cat# 22665-1-AP), was performed to confirm the identity of the isolated cells as GCs.

### Immunohistochemistry

Tissues were excised and immediately fixed in 4% paraformaldehyde. Sections were prepared and underwent immunohistochemical staining using the TRIM21 antibody (Cell Signaling Technology, cat# 92043) and CPT1A antibody (Cell Signaling Technology, cat# 97361), according to the standard procedures of the immunohistochemistry kit (KeyGEN, cat# KGC3201-300). Stained slides were examined with a microscope (Nikon, ci-L). The H-score system was employed for semiquantitative analysis of staining results. The H-score was calculated using the formula: Σ(PI × *I*), where PI represents the percentage of positive cells and *I* represents the staining intensity. Staining intensity was scored as follows: 0 (no staining), 1 (weak staining/light yellow), 2 (moderate staining/brownish yellow), and 3 (strong staining/dark brown). Five random fields per sample were selected under 200× magnification for scoring, and the average value was taken as the final H-score of that sample (score range: 0 to 300).

### Human ovarian GC extraction

A total of 10 Chinese women were enrolled from outpatients who sought assisted reproduction technology treatment at the Reproductive Medicine Center, Shengjing Hospital, China Medical University. This study included 5 women diagnosed with PCOS and 5 healthy women as controls. PCOS diagnosis was established according to the 2003 Rotterdam criteria, requiring at least 2 of the following features after exclusion of other etiologies: oligo-ovulation or anovulation; clinical or biochemical hyperandrogenism; polycystic ovarian morphology on ultrasound. Human GCs were isolated and purified from PCOS patients and controls. On the day of oocyte retrieval, cumulus cells tightly surrounding the oocytes were transferred together with the oocytes for subsequent preservation. Follicular fluid from large follicles (>14 mm) containing parietal GCs was then collected and centrifuged (1,500 × g, 15 min), collecting the precipitate. The precipitate was then transferred to lymphocyte separation medium (Lymphoprep) and centrifuged (3,000 × g, 10 min). After separation from the interlaminar phase, the GCs were washed with PBS for subsequent experiments such as RT-quantitative PCR.

### Cell culture and treatment

Human granulosa-like tumor cell lines KGN and COV434 were obtained from Suzhou Haixing Biosciences Co., Ltd. KGN cells were cultured in Dulbecco's modified Eagle medium (KeyGEN, cat# KGL1211-500) and COV434 cells in RPMI 1640 medium (KeyGEN, cat# KGL1503-500), both supplemented with 10% fetal bovine serum and 1% penicillin–streptomycin, and incubated at 37 °C in a humidified atmosphere with 5% CO_2_.

Cells were exposed to 100 nM DHT for 72 h. To investigate specific cellular processes, cells were also subjected to various inhibitors: 100 nM MLN4924 (Selleck, cat# S7109) for 24 h to inhibit neddylation; 5 μM MG132 (Selleck, cat# S2619) for 24 h to inhibit proteasomal degradation; 500 nM CHX (MCE, cat# HY-12320) for 4 h to inhibit protein synthesis; and 50 μM etomoxir (MCE, cat# HY-50202) for 24 h to inhibit FAO. Dimethyl sulfoxide (Sigma-Aldrich, cat# D5879) served as the vehicle control for all cell cultures.

### Plasmids and stable cell line construction

Target cDNA sequences were PCR-amplified and cloned into the pcDNA3.1 backbone. Specifically, UBE2M, TRIM21, CPT1A, and ubiquitin were ligated into pcDNA3.1-Flag, pcDNA3.1-MYC, pcDNA3.1-Flag, and pcDNA3.1-HA expression vectors, respectively. The Flag-CPT1A (K148R) and Flag-CPT1A (K161R) sequences were derived from the pcDNA3.1-Flag-CPT1A plasmid. Furthermore, shRNA vectors targeting UBE2M, TRIM21, CPT1A, and NEDD8 were constructed by inserting the corresponding human small interfering RNA sequences into the PLKO.1 puro lentiviral shRNA vector: UBE2M no.1, 5′-CAGAGGUCCUGCAGAACAATT-3; UBE2M no.2, 5′-UUGUUCUGCAGGACCUCUGTT-3′; TRIM21 no. 1, 5′-AGGUAGAUGUGACAGGAAATT-3′ ; TRIM21 no. 2, 5′-GCACAGGAGUACCAGGAGATT-3′; CPT1A no.1, 5′-CCGCAAAUCUUCUGGCAAATT-3′; CPT1A no.2, 5′-CCAUGAAGCUCUUUAGACAATT-3′; NEDD8, 5′-GAGAUUGAGAUAGACAUCGAATT-3′. In vitro transfection of KGN cells with plasmids and lentiviruses was carried out using Invitrogen (KeyGEN, cat# KGA9705-1.5) according to the manufacturer’s protocol. Forty-eight hours after the transfection, cells were collected for screening or experimental procedures. All plasmids and lentiviral shRNAs were sourced from Nanjing Zebrafish Biotech Co., Ltd.

### Western blotting

Cell or tissue samples were lysed in radioimmunoprecipitation assay buffer (KeyGEN, cat# KGB5203-100) with protease and phosphatase inhibitors. Protein concentration was measured by bicinchoninic acid assay (SEVEN, cat# SW201-02). Equal amounts of protein (20 μg per lane) were resolved by sodium dodecyl sulfate-polyacrylamide gel electrophoresis and subsequently blotted onto a polyvinylidene fluoride membrane (Cytiva). After blocking in 5% nonfat milk for 1 h, membranes were incubated overnight at 4 °C with primary antibodies and then with secondary antibodies for 1 h at ambient temperature. Signals were developed with ECL reagent (SEVEN, cat# SW134-01) and imaged with a chemiluminescent detection system (GE AI800, USA). Western blot quantification was performed using ImageJ, with normalization to loading controls. Primary antibodies were used as follows: anti-TRIM21 (Cell Signaling Technology, cat# 92043), anti-TRIM21 (Proteintech, cat# 12108-1-AP), anti-TRIM21 (Proteintech, cat# 67136-1-Ig), anti-CPT1A (Cell Signaling Technology, cat# 12252), anti- CPT1A (Cell Signaling Technology, cat# 97361), anti-CPT1A (Proteintech, cat# 66039-1-Ig), anti-CPT1A (Starter, cat# S0B0972), anti-UBE2M (Abcam, cat# ab109507), anti-NEDD8 (Abcam, cat# ab81264), anti-NEDD8 (Proteintech, cat# 16777-1-AP), anti-ubiquitin (Cell Signaling Technology, cat# 58395), anti-ubiquitin K48 (Cell Signaling Technology, cat# 8081), anti-ubiquitin K48 (Cell Signaling Technology, cat# 5621), anti-α-tubulin (Cell Signaling Technology, cat# 5059), anti-ATP5A1 (Proteintech, cat# 14676-1-AP), anti-UQCRC2 (Selleck, cat# F1592), anti-MTCO1 (Selleck, cat# F1682), anti-SDHB (Selleck, cat# A5786), anti-NDUFB8 (HUABIO, cat# ET7108-25); anti-hemagglutinin (Cell Signaling Technology, cat# 2367), anti-Flag (Proteintech, cat# 20543-1-AP), anti-Myc (HUABIO, cat# R1208-1); anti-glyceraldehyde-3-phosphate dehydrogenase (Cell Signaling Technology, cat#2118), anti-beta actin (HUABIO, cat# EM21002); horseradish peroxidase-conjugated Goat Anti-Rabbit IgG (H+L) (Proteintech, cat# SA00001-2), horseradish peroxidase-conjugated Goat Anti-Mouse IgG (H+L) (Proteintech, cat# SA00001-1).

### RNA isolation and quantitative PCR

Total RNA was extracted with TRIzol reagent (Thermo Fisher Scientific, cat# 15596026) and an RNA isolation kit (Vazyme, cat# RC102-01, cat# RC112-01) following the manufacturers’ protocols. The extracted RNA was reverse transcribed to cDNA using the PrimeScript RT Reagent Kit with gDNA Eraser (Takara, cat# RR047A). Quantitative PCR was performed on an Applied Biosystems Quant Studio 5 system (Thermo Fisher Scientific, A28575) using SYBR Green Master Mix (Takara, cat# RR820), with glyceraldehyde-3-phosphate dehydrogenase (*GAPDH*) serving as the internal reference. Primer sequences are provided in Table S6.

### Immunofluorescence staining

Cells were plated on glass coverslips positioned in 24-well plates and cultured under standard humidified conditions. Upon reaching desired confluence, cells were fixed with 4% paraformaldehyde (Beyotime, cat# P0099) for 5 min, followed by permeabilization using 0.2% Triton X-100 (Solarbio, cat# T8200) for 5 min at room temperature. Nonspecific binding was saturated with 5% BSA (SEVEN, cat# SW127-03). Next, primary antibodies (UBE2M, TRIM21, CPT1A, and NEDD8) were applied and allowed to bind overnight at 4 °C. After thorough rinsing with PBS (KeyGEN, cat# KGC4307-500), fluorescently labeled secondary antibodies were added for 1 h at ambient temperature. Nuclei were counterstained with 4′,6-diamidino-2-phenylindole (KeyGEN, cat# KGA1529-25).

### Co-IP and ubiquitination assays

Cells were harvested at the indicated time posttransfection and lysed using IP buffer (1 % Triton X-100, 10 % glycerol, 1 M tris-HCl, 150 mM NaCl, 50 mM EDTA, and protease inhibitors, pH 7.4). After centrifugation, supernatants were incubated with protein A/G magnetic beads (Selleck, cat. B23202) plus respective antibodies for overnight rotation at 4 °C. After triple washing with IP buffer, the captured proteins and their ubiquitination status were assessed by western blot.

### Measurement of mitochondrial membrane potential

Mitochondrial membrane potential was evaluated using TMRE (MCE, cat# HY-D0985A) and JC-1 (Beyotime, cat# C2006). Mitochondria and nuclei were labeled with MitoTracker Green (MTG, Beyotime, cat# C1048) and Hoechst (Servicebio, cat# G1011), respectively. Cellular samples were incubated with either 100 μM TMRE or JC-1 working solution for 15 to 30 min at 37 °C, protected from light. Colocalization analysis was carried out using a laser scanning confocal microscope (Nikon, A1, JAPAN). TMRE was excited at 549 nm, and emission signals was collected using a 575-nm band-pass filter. For JC-1, excitation was performed at 488 nm, and emission signals were captured using a 590-nm band-pass filter. Images were processed using ImageJ software.

### Seahorse analysis

OCR and ECAR were detected using a Seahorse XF analyzer (XFe96; Agilent Technology). The seeding density of 8,000 cells per well was optimized through preliminary experiments to ensure that cells reached approximately 80% to 90% confluence at the time of assay, thereby maintaining metabolic activity within the linear detection range while avoiding overconfluence or stress-induced artifacts. Treated cells were further plated in 200 μl of medium in an XF96 cell culture microplate and incubated for 14 to 16 h at 37 °C in a humidified 5% CO_2_ atmosphere. Culture medium was exchanged for XF medium 1 h premeasurement. For OCR, sequential injections after baseline recording included 1.5 μM oligomycin (ATP synthase inhibitor), 1.5 μM FCCP (mitochondrial uncoupler), and 0.5 μM rotenone/0.5 μM antimycin A (mitochondrial electron transport chain inhibitors). Mitochondrial function indices, including basal respiration, maximal respiration, ATP production, and coupling efficiency, were subsequently calculated. For ECAR, after baseline measurements, 10 mM glucose (basal glycolysis assessment), 1 μM oligomycin (maximal glycolytic capacity), and 50 mM 2-deoxy-d-glucose (nonglycolytic acidification determination) were sequentially employed. Glycolytic profiles, including glycolysis and glycolytic capacity, were determined. Data were processed using the Seahorse XFe96 software (Seahorse Bioscience, Agilent).

### Mitochondrial respiratory chain enzyme activity

Mitochondrial complexes I to V activities were quantified in cell extracts by single-wavelength spectrophotometry using kits (Solarbio, cat. #BC0510, BC3240, BC0945, and BC1440) per the manufacturer’s instructions. Cell lysates (5 × 10^6^ cells) underwent sequential centrifugation at 600 × g for 10 min and 11,000 × g for 15 min at 4 °C; the pelleted fraction was sonicated and incubated with the corresponding substrates to initiate each complex’s enzymatic reaction The metabolic product was recorded on a spectrophotometer (Agilent Technology, USA)

### Transmission electron microscopy

Ovaries from mice underwent glutaraldehyde (3%, 2 h) and osmium tetroxide (2%, 2 h) fixation, ethanol dehydration, and embedding and then sectioned to approximately 70-nm thickness and subjected to contrasting with uranyl acetate and lead citrate staining. Mitochondrial ultrastructure in oocytes and adjacent GCs of secondary follicles was examined by TEM (JEM2000EX, JEOL, Sagamihara, Japan). Mitochondrial number and morphology were quantified in each micrograph.

### Molecular docking

Spatial structures of UBE2M, TRIM21, and CPT1A were obtained from the UniProt database. The spatial binding characteristics and potential interactions between each protein pair were modeled using the HDOCKlite v1.1 molecular docking platform system. Binding reliability of the resulting complexes was assessed by docking score, confidence score, and ligand root mean square deviation, where a docking score below −100 signifies high binding affinity and a confidence score above 0.7 indicates robust model credibility.

### MicroScale thermophoresis

KGN cells were transfected with green fluorescent protein-CPT1A plasmid (with green fluorescent protein alone as control) and lysed using IP buffer (50 mM tris, 150 mM NaCl, and 0.3% Triton X-100, pH 7.5). Recombinant human TRIM21 was serially diluted in PBST (PBS with 0.1% Tween 20) from 200 μM to approximately 1.5 nM and mixed with clarified lysates, then incubated in darkness at ambient temperature for 20 min. Protein–ligand interactions were measured on a Monolith NT.115 instrument (NanoTemper Technologies), and binding curves from 3 independent experiments were fitted using NT analysis software (MO. Affinity Analysis) and visualized in GraphPad Prism 9.0.

### Oocyte collection and maturation

Female C57BL/6J mice (6 to 8 weeks old) received an intraperitoneal administration of 10 IU of PMSG and were sacrificed 48 h later. After ovary dissection and removing surrounding adipose tissue, germinal vesicle-stage oocytes were isolated and maintained at M16 medium (Sigma-Aldrich, cat# M7292) at 37 °C in 5% CO₂ for 14 h to achieve IVM. Only oocytes with intact germinal vesicles, homogeneous cytoplasm, and compact cumulus cell layers were selected for subsequent experiments.

To collect ovulated oocytes, female C57BL/6J mice were primed with 10 IU of PMSG and then administered 10 IU of human chorionic gonadotropin (hCG) (Ningbo Second Hormone Factory) 48 h later. Oocytes were harvested from oviduct ampullae 13 to 14 h post-hCG, and cumulus cells were gently dispersed via a short exposure to hyaluronidase (Sigma-Aldrich, cat# H3884-50MG).

### In vitro fertilization and embryo in vitro culture

Superovulation was stimulated by intraperitoneal administration of 10 IU of PMSG, followed 48 h later by 10 IU of human hCG. Fourteen to 16 h after hCG administration, metaphase II (MII) oocytes were harvested from the ampullae of the oviduct and transferred to human tubal fluid (HTF) medium (Biorigin, cat# BN80001). Adult C57BL/6J male epididymal sperm were capacitated in Toyoda–Yokoyama–Hoshi medium (Biorigin, cat# BN80002) for 1 h at 37 °C with 5% CO₂, and 3 μl of the resulting suspension was added to HTF containing MII oocytes for in vitro fertilization at 37 °C with 5% CO₂. Six hours after fertilization, presumptive zygotes were washed 3 times in HTF and cultured overnight under mineral oil (Sigma-Aldrich, cat# M8410-1L). Two-cell embryos were then transferred to potassium simplex optimized medium (KSOM) medium (MesGen Biotech, cat# MKS1050A) and cultured at 37 °C for 4 d.

### Evaluation of spindle morphology

MII oocytes underwent fixation in 4% paraformaldehyde (Beyotime, cat# P0099) for 30 min, permeabilization in PBS (KeyGEN, cat# KGC4307-500) supplemented with 0.5% Triton X-100 (Solarbio, cat# T8200) for 40 min at ambient temperature, and blockage in PBS with 3% BSA (SEVEN, cat# SW127-03) for 30 min. After overnight incubation with α-tubulin antibody (Cell Signaling Technology, cat#5059), oocytes were washed 3 times in 0.1% PBST and stained with 4′,6-diamidino-2-phenylindole for 10 min. Finally, oocytes were mounted in PBS droplets on glass-bottom dishes for imaging.

### Oocyte lipid droplet staining

Lipid content was assessed via BODIPY 493/503 (MCE, cat# HYW090090). Oocytes were isolated under a stereomicroscope with a micromanipulation pipette, transferred to PBS, and rinsed until the background was free of debris. They were then incubated in a 5 μM working solution at 37 °C in subdued light for 20 min, washed 3 times in culture medium, and finally transferred to confocal dishes for imaging.

### Transcriptome data analysis

Total RNA was extracted from mouse ovarian tissues, and libraries were prepared using the Illumina standard instruction (VAHTS Universal V6 RNA-seq Library Prep Kit for Illumina). Prior to sequencing on an Illumina NovaSeq 6000, library integrity and fragment-length profiles were verified on an Agilent 4200 Bioanalyzer. Raw reads were processed with Seqtk before mapping to genome via Hisat2 (v2.0.4) [62]. Gene fragments were calculated using stringtie (v1.3.3b) and normalized via the trimmed mean of *M* values method [63–66]. Differentially expressed genes were classified as those exhibiting a false discovery rate (FDR) < 0.05 and a fold change > 2, as determined by edgeR [66,67]. Primer sequences are listed in Table S6.

### Quantitative global proteomic analysis based on the 4D Fast DIA technique

Quantitative proteomic profiling was carried out using the 4D Fast data-independent acquisition (DIA) workflow: Samples were prepared by protein extraction and trypsin digestion and analyzed by liquid chromatography-tandem mass spectrometry, and the resulting DIA data were processed in Spectronaut (v.18) software with database searching at a protein-, peptide-, and PSM-level FDR < 1%. Relative differential proteomic and downstream bioinformatics analyses were then conducted using a significance threshold of *P* < 0.05 and a fold change cutoff of 1.5.

### Functional enrichment analysis

Functional enrichment of differentially expressed genes was assessed by GO and Kyoto Encyclopedia of Genes and Genomes analyses. GO analysis classified gene functions into 3 categories: biological processes, molecular functions, and cellular components [68], and Kyoto Encyclopedia of Genes and Genomes analysis identified associated pathways, diseases, and drug interactions [69]. Both analyses were performed via clusterProfiler R package, with a FDR < 0.05 considered significant.

### IP-MS

For mass spectrometry analysis of TRIM21-binding proteins, KGN cells were transfected with Myc-TRIM21 for 24 h, then supplemented with 5 μM MG132 for 4 h, and subsequently immunoprecipitated using anti-Myc magnetic beads. To identify ubiquitination sites on CPT1A, KGN cells were transfected with Flag-CPT1A for 24 h, followed by immunoprecipitation of cell lysates with anti-Flag magnetic beads. The immunoprecipitated proteins underwent extraction, trypsin digestion, and liquid chromatography-mass spectrometry analysis. DIA data processing was performed using the DIA-NN search engine (v.1.8) with searches against the Homo_sapiens_9606_SP_20231220.fasta (20429 entries) and a reverse decoy database. Trypsin/P is designated as the cleavage enzyme, allowing for a maximum of 1 cleavage site deletion, with fixed modifications including N-terminal methionine excision and cysteine carbamidomethylation. The FDR threshold was set to 1%.

### Statistical analysis

All experiments were performed in at least 3 independent biological replicates. While investigators directly performing the experiments were not blinded to group allocation, statistical analyses were conducted independently. Data are presented as means ± SD or standard error of the mean. Statistical analyses and graphical presentations were conducted using GraphPad Prism 9.0. One-way analysis of variance (ANOVA) followed by Tukey’s or Bonferroni multiple comparison post hoc correction, or unpaired 2-tailed Student *t* test, were used for group comparisons. Blood glucose analysis between groups was performed by 2-way ANOVA and multiple comparison test.

## Ethical Approval

This study was approved by the Ethics Committee of Shengjing Hospital, China Medical University on 2025 June 17 (Approval No. 2025PS014F). Informed consent was obtained from all participants before inclusion in the study. All animal procedures were approved by the Animal Care and Use Committee of Shengjing Hospital, China Medical University (Approval No. 2024PS001K) and strictly adhered to the National Institutes of Health guidelines for the care and use of laboratory animals.

## Data Availability

The processed and raw data, as well as the scripts used to generate the figures, are available upon reasonable request from the corresponding authors.
