# Supplementary material for: Inhibiting TRIM21 Neddylation Rejuvenates Oocyte Quality in PCOS by Regulating Ubiquitination of CPT1A
Source: Research (Wash D C). 2026 Apr 3;9:1223. doi: 10.34133/research.1223 (PMC13047273; doi:10.34133/research.1223)
Supplement: Supplementary 1 — Figs. S1 to S7 Tables S1 to S6 [file research.1223.f1.zip › Supplementary Figure Legends.pdf]

Supplementary Figure Legends

Fig.S1: High expression of TRIM21 in ovarian granulosa cells of PCOS mice, related to Figure 1.

(A) vaginal smears at different stages of the estrus cycle. P, proestrus; E, estrus; M, metestrus; D, diestrus (scale bars, 100μm). (B) body weight of adult female mice (n=15). (C) Morphology of pups and implantation site in the uterus of mice (white arrow). (D) Schematic illustration showing PCOS induced by intraperitoneal injection of DHT. (E) Estrous cycle of adult female mice for 21 consecutive days (n=5). (F) GTT test after 16 hours of fasting and ITT test after 4 hours of fasting in adult female mice (n=5).

(G) Anogenital distance in adult female mice (n=15). Data are expressed as mean ± SEM, and each symbol represents biologically independent mice. Significance was calculated by one-way ANOVA multiple comparison test. Blood glucose analysis between groups (F) was determined by two-way ANOVA and multiple comparison test. NS, not significant; *$p < 0.05$; **$p < 0.01$; ***$p < 0.001$; ****$p < 0.0001$.

Fig.S2: High expression of TRIM21 in ovarian granulosa cells of PCOS mice, related to Figure 1.

(A and B) Testosterone and LH levels of serum (n=15). (C) body weight of adult female mice (n=15). (D) H&E staining of ovaries showing cystic follicle (*), and corpora lutea (#; scale bars, 100μm). (E) RT-PCR analysis of TRIM21 mRNA levels in mice ovaries. (F) WB analysis of TRIM21 in mice ovaries. (G) FSHR Immunofluorescence

verification image of mice primary granulosa cells. (H) H-score analysis of TRIM21 in mice ovarian tissues(n=5). (I) RT-PCR analysis of TRIM21 in human follicular fluid(n=5). Data are expressed as mean ± SD and each symbol represent biologically independent simple. ns, not significant; *p < 0.05; **p < 0.01; ***p < 0.001; ****p < 0.0001.

Fig.S3: TRIM21 is associated with fatty acid oxidation in ovarian granulosa cell, related to Figure 2.

(A and B) Evaluation of the mitochondrial OXPHOS function by OCR in KGN cell (n=6). (C to E) Glycolytic function assay by OCR in KGN cell, including baseline and glycolytic capacity (n=3). (F) Activity of mitochondrial complex I-V in KGN cell (n=6). (G) WB analysis of OXPHOS subunits I-V in COV434 cell. Data are expressed as mean ± SD. *p < 0.05; **p < 0.01; ***p < 0.001; ****p < 0.0001.

Fig.S4: TRIM21 regulates fatty acid oxidation in granulosa cells through CPT1A, related to Figure 3.

(A) Co-IP and WB analysis of TRIM21and CPT1A of KGN cell. (B and C) WB analysis of CPT1A with TRIM21 silencing or overexpression of COV434 cell. (D to K) RT-PCR analysis of mRNA levels of follicle development-related genes in mouse COCs (n=5). Data are expressed as mean ± SD. *p < 0.05; **p < 0.01; ***p < 0.001; ****p < 0.0001.

Fig.S5: UBE2M promotes TRIM21 neddylation and its interaction with CPT1, related to Figures 4 and 5.

(A) WB analysis of the effect of BafA1 on CPT1A in KGN cells with TRIM21 overexpression. (B) In KGN cell, Co-IP and WB of exogenous ubiquitination of CPT1A treated with Flag-CPT1A mutant plasmids in the presence of MG132 (10μM, 4h).(C and D) Evaluation of the mitochondrial OXPHOS function by OCR, including basal respiration and maximum respiration (n=6). (E and F) WB analysis of cell lysates immunoprecipitated with UBE2M or TRIM21 antibodies of KGN cell. (G) WB analysis of NEDD8 after knockdown of TRIM21 in KGN cell. (H) Co-IP and WB analysis of TRIM21 neddylation with or without UBE2M overexpression in KGN cell. (I) WB analysis of CPT1A treated with UBE2M overexpression, with or without TRIM21 knockdown in KGN cell. (J) Co-IP and WB analysis of KGN cell lysates immunoprecipitated with anti-MYC antibody in the presence of UBE2M overexpression.(K) WB analysis of CPT1A treated with different concentrations of MLN4924 in KGN cell.

Fig.S6: MLN4924 reverses the ubiquitination of CPT1A by TRIM21 and ameliorates the phenotype of PCOS mice, related to Figure 6.

(A and B) Evaluation of the glycolysis function by ECAR in KGN cell, including baseline and glycolytic capacity (n=3). (C) Activity of mitochondrial complexes I-IV in KGN cells (n=6). (D) Estrous cycle of PAMH F1 adult female mice for 21 consecutive days (n=15). (E) GTT test of PAMH F1 fasted for 16 hours (n=8). (F)

Estrous cycle of DHT adult female mice for 21 consecutive days (n=15). (G and H) Testosterone and LH levels of serum (n=15). (I and J) GTT and ITT after 16 hours of fasting and 4 hours of fasting in DHT mice (n=8). Data are expressed as mean ± SD, and each symbol represents biologically independent mice. Significance was calculated by one-way ANOVA multiple comparison test. Blood glucose analysis between groups(E, I and J) was determined by two-way ANOVA and multiple comparison test.*$p < 0.05$; **$p < 0.01$; ***$p < 0.001$; ****$p < 0.0001$.

Fig.S7: MLN4924 reverses the ubiquitination of CPT1A by TRIM21 and ameliorates the phenotype of PCOS mice, related to Figures 6 and 7.

(A) Body weight of adult female mice (n=15). (B) Number of pups per birth (n=10). (C) WB analysis of TRIM21 and CPT1A in mice ovarian GCs. (D) RT-PCR analysis of TRIM21 and CPT1A mRNA levels in mice ovarian GCs. (E) H-score analysis of TRIM21 in mice ovarian tissues (n=5). (F) RT-PCR analysis of mRNA levels of follicle development-related genes in mouse COCs (n=5). Data are expressed as mean ± SD, and each symbol represents biologically independent mice. *$p < 0.05$; **$p < 0.01$; ***$p < 0.001$; ****$p < 0.0001$.
