# Supplementary figures and images for: Inhibiting TRIM21 Neddylation Rejuvenates Oocyte Quality in PCOS by Regulating Ubiquitination of CPT1A
Source: Research (Wash D C). 2026 Apr 3;9:1223. doi: 10.34133/research.1223 (PMC13047273; doi:10.34133/research.1223)

Fig S1

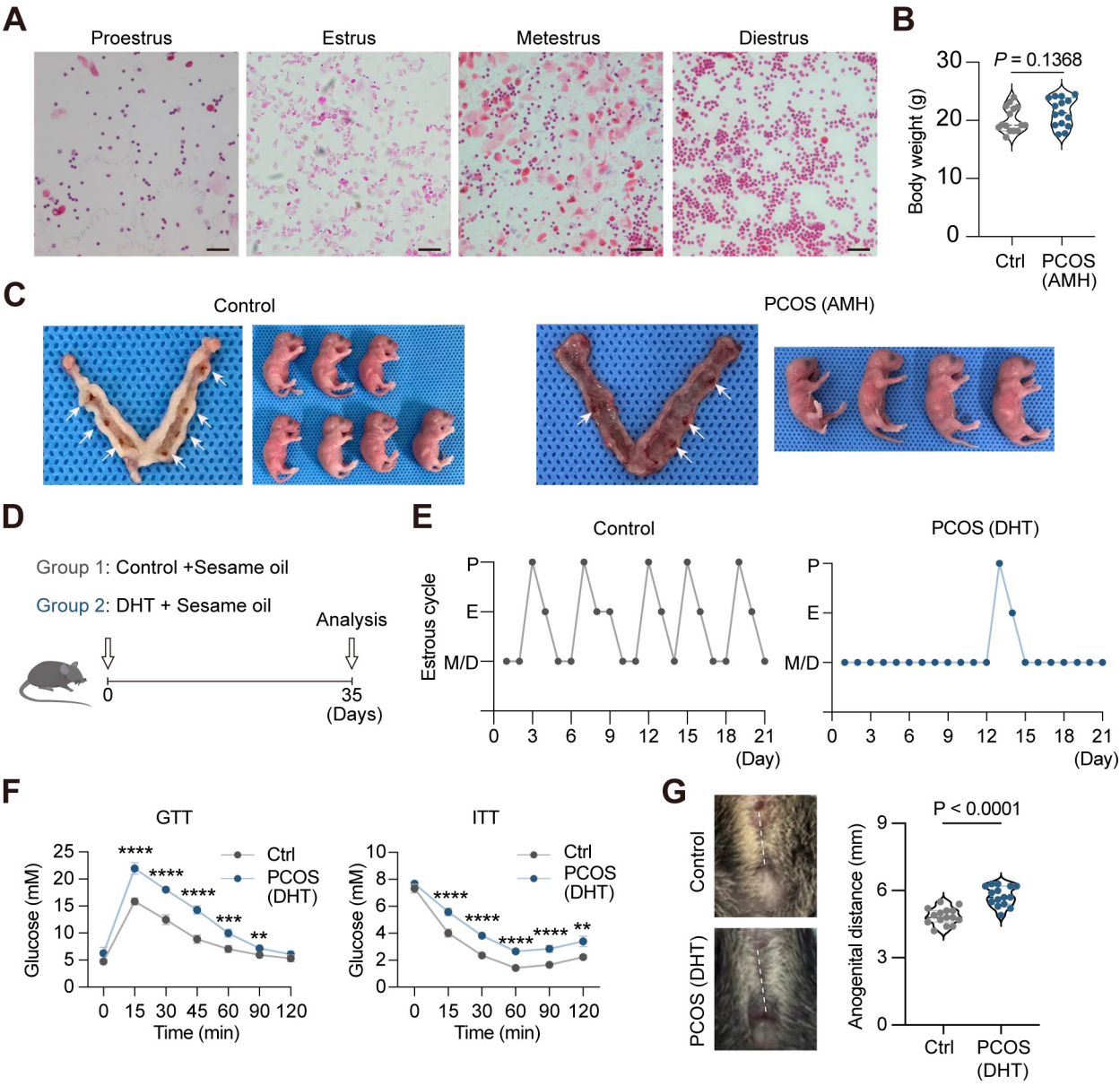

Supplement: Supplementary 1 — Figs. S1 to S7 Tables S1 to S6 [file research.1223.f1.zip › Figure S1.pdf]

Fig S2

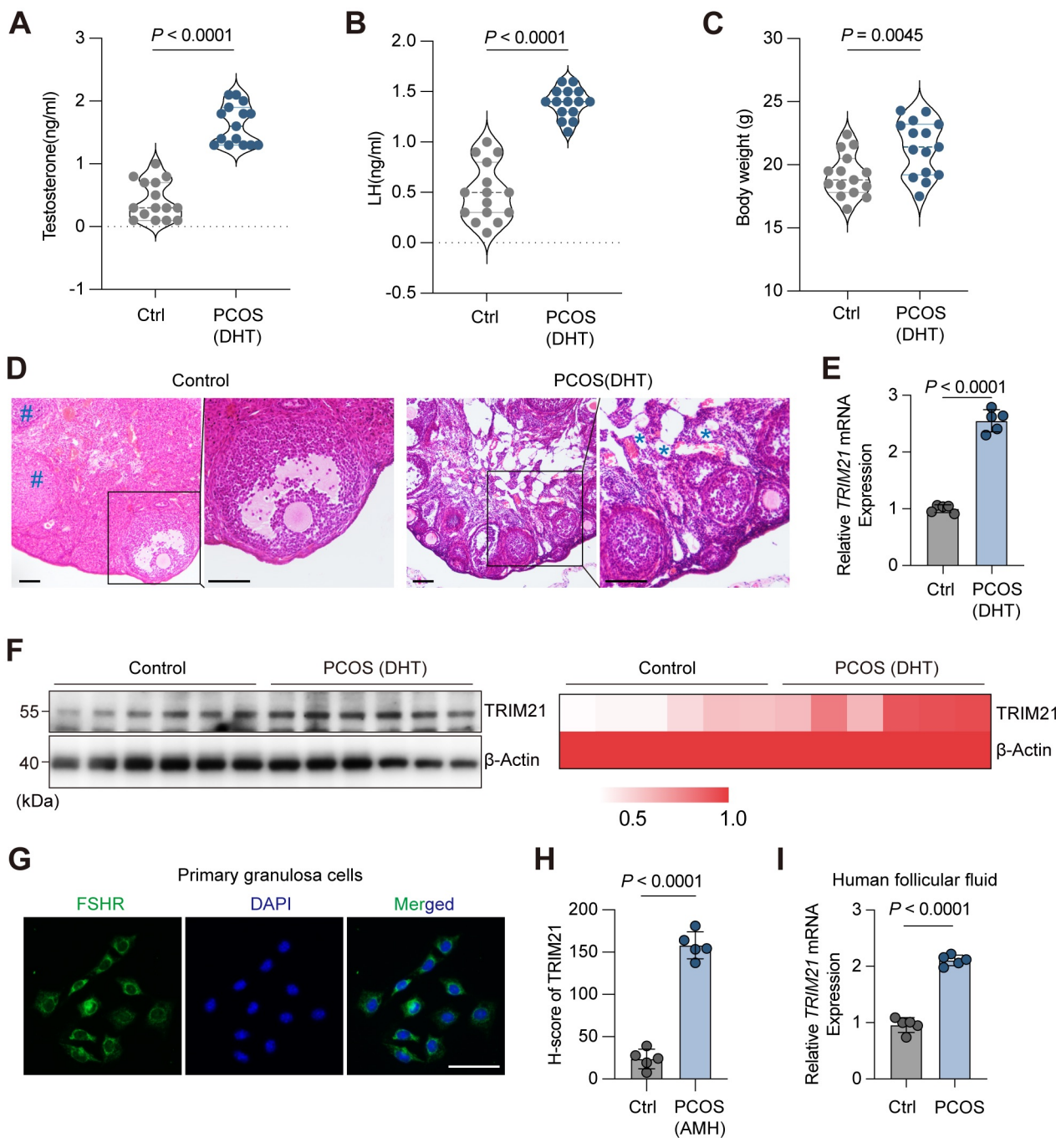

Supplement: Supplementary 1 — Figs. S1 to S7 Tables S1 to S6 [file research.1223.f1.zip › Figure S2.pdf]

Fig S3

**A**

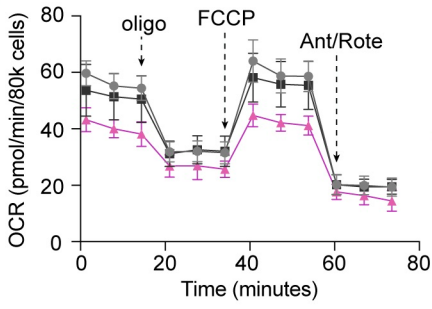

**B**

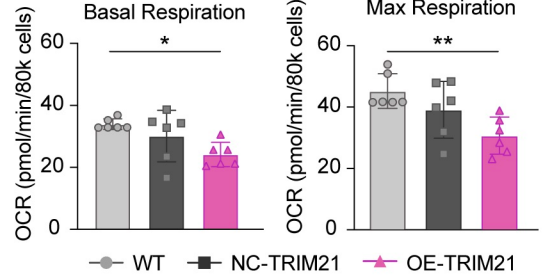

**C**

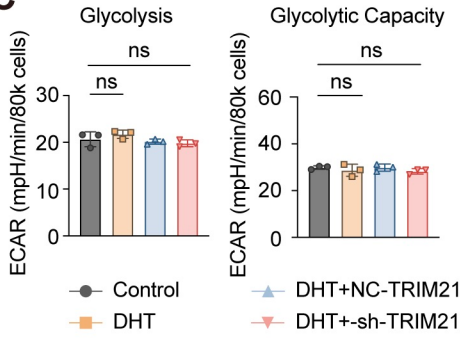

**D**

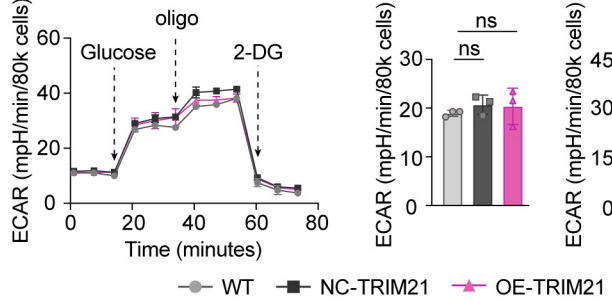

**E**

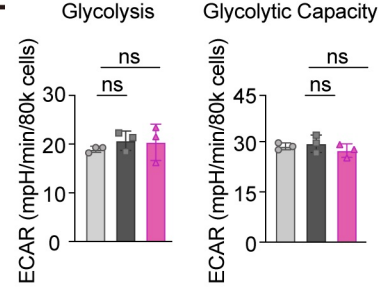

**F**

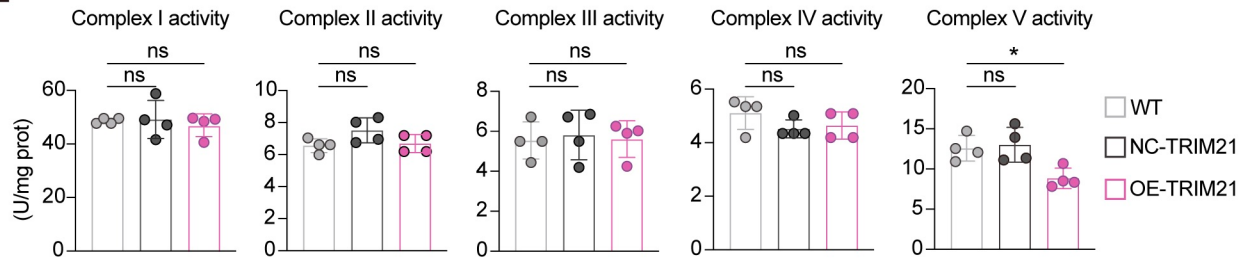

**G**

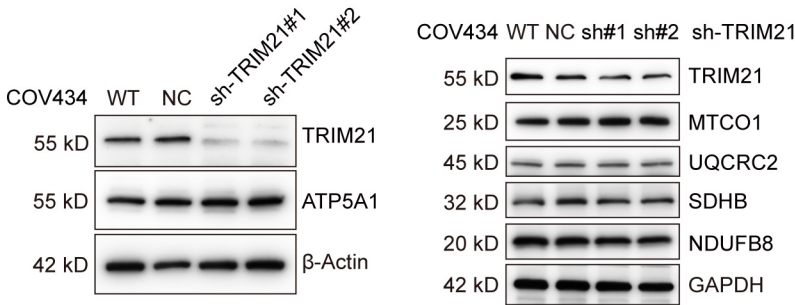

Supplement: Supplementary 1 — Figs. S1 to S7 Tables S1 to S6 [file research.1223.f1.zip › Figure S3.pdf]

Fig S4

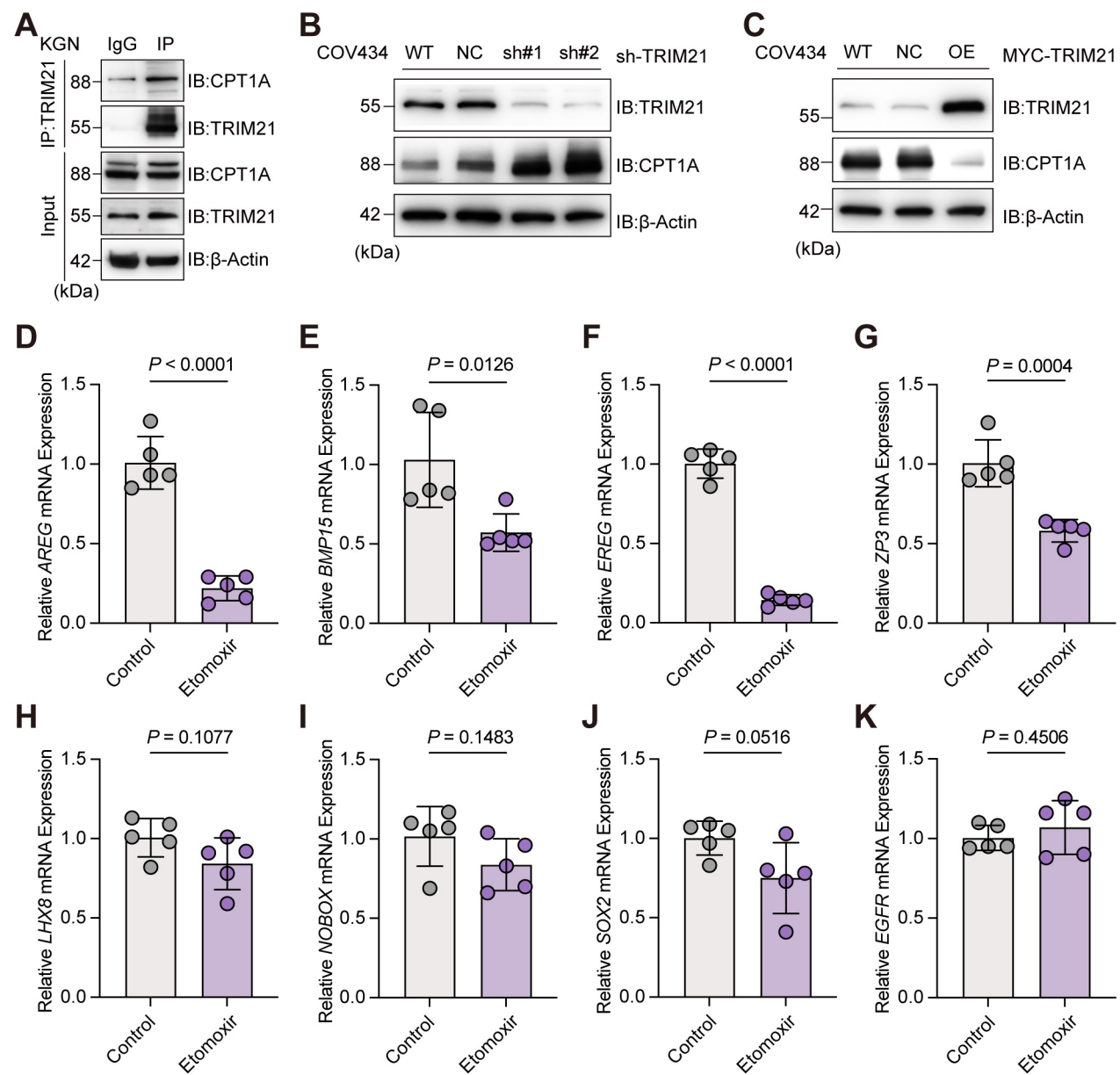

Supplement: Supplementary 1 — Figs. S1 to S7 Tables S1 to S6 [file research.1223.f1.zip › Figure S4.pdf]

# Fig S5

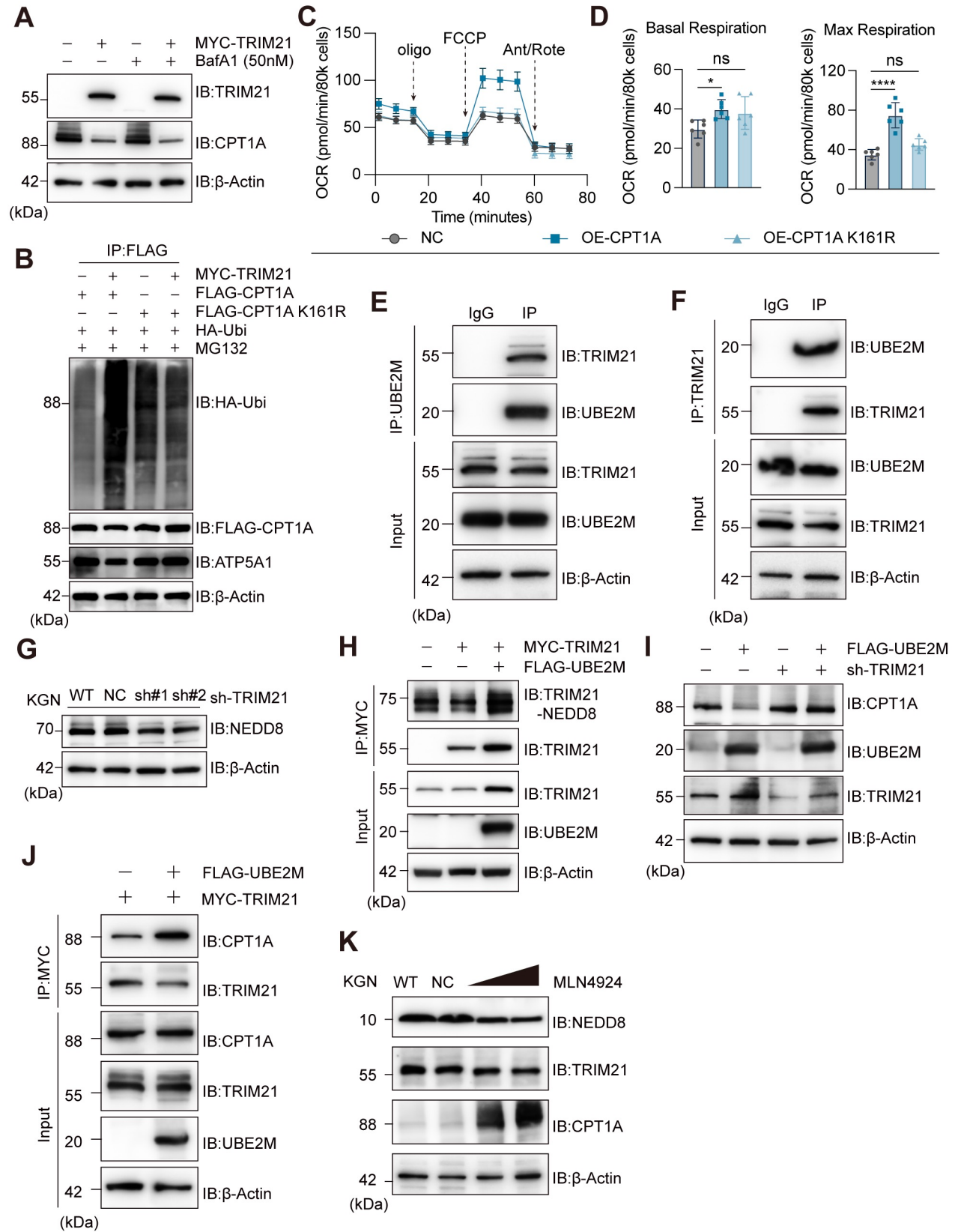

Supplement: Supplementary 1 — Figs. S1 to S7 Tables S1 to S6 [file research.1223.f1.zip › Figure S5.pdf]

Fig S6

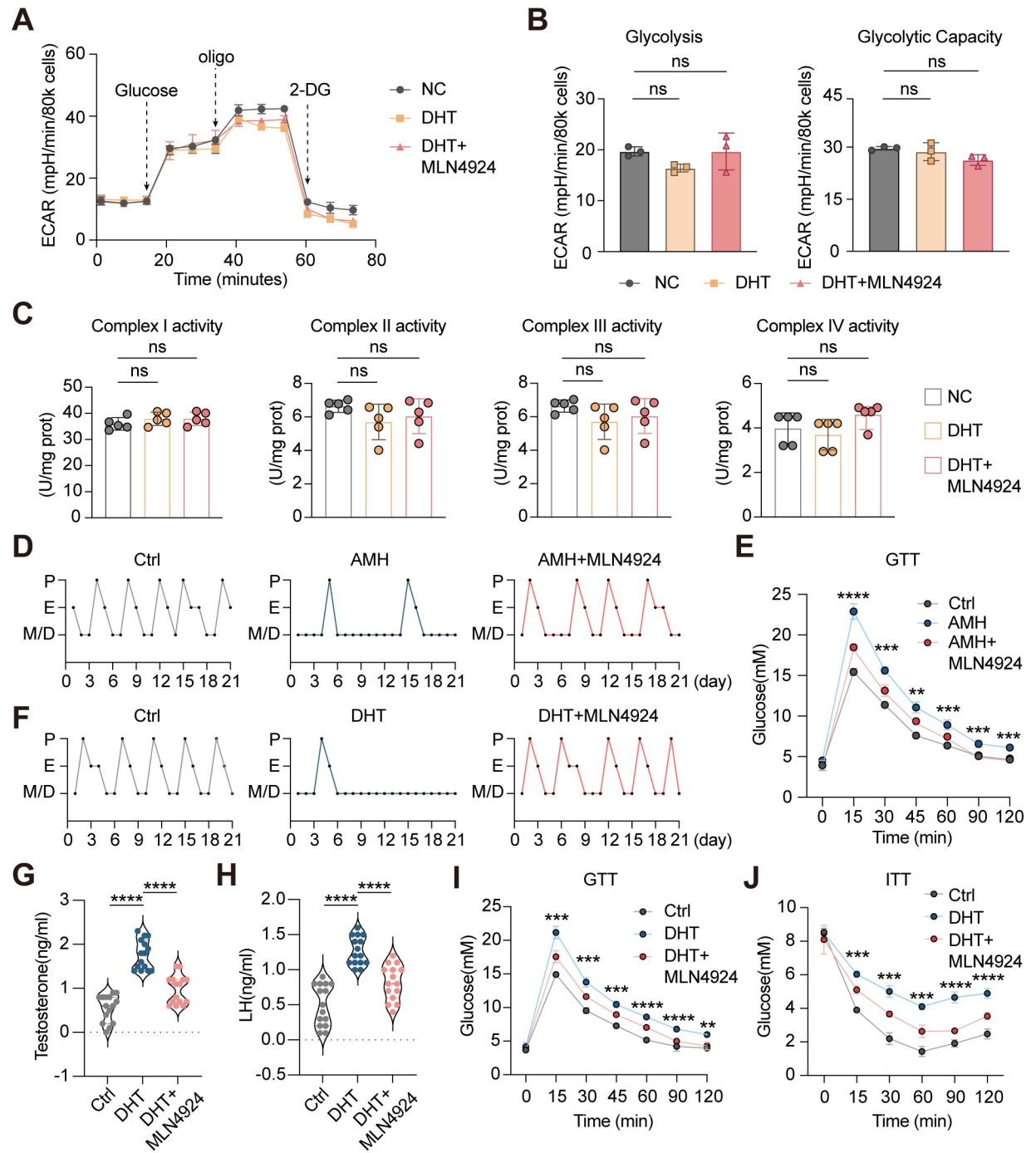

Supplement: Supplementary 1 — Figs. S1 to S7 Tables S1 to S6 [file research.1223.f1.zip › Figure S6.pdf]

Fig S7

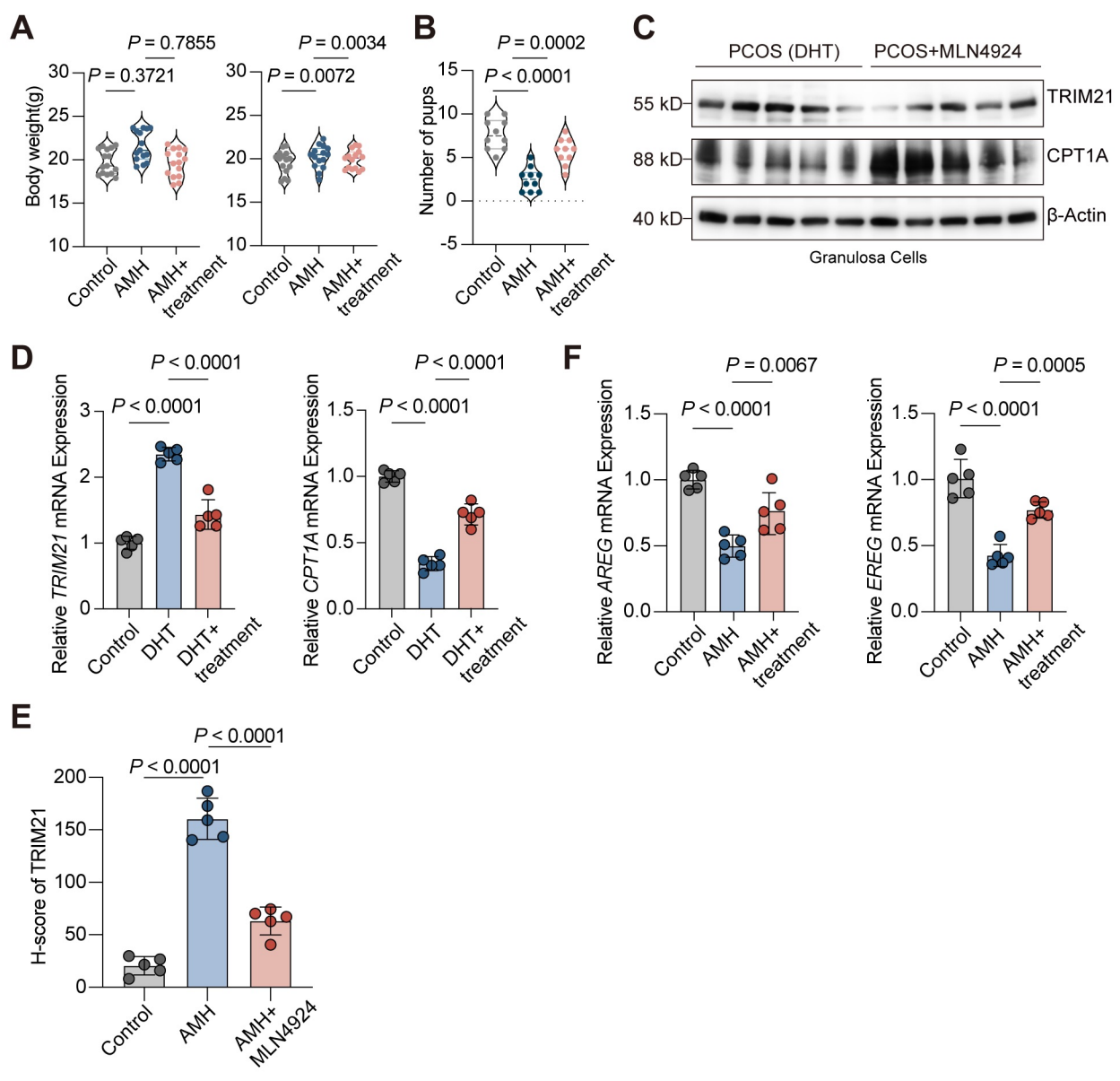

Supplement: Supplementary 1 — Figs. S1 to S7 Tables S1 to S6 [file research.1223.f1.zip › Figure S7.pdf]
